# Internalization-Dependent Free Fatty Acid Receptor 2 Signaling Is Essential for Propionate-Induced Anorectic Gut Hormone Release

**DOI:** 10.1016/j.isci.2020.101449

**Published:** 2020-08-12

**Authors:** Natarin Caengprasath, Noemi Gonzalez-Abuin, Maria Shchepinova, Yue Ma, Asuka Inoue, Edward W. Tate, Gary Frost, Aylin C. Hanyaloglu

**Affiliations:** 1 Institute of Reproductive and Developmental Biology (IRDB), Department of Metabolism, Digestion and Reproduction, Imperial College London, Rm 2009, Hammersmith Campus, Du Cane Road, London W12 0NN, UK; 2 Department of Metabolism, Digestion and Reproduction, Imperial College London, London, UK; 3 Department of Chemistry, Imperial College London, London, UK; 4 Graduate School of Pharmaceutical Sciences, Tohoku University, Sendai, Japan

**Keywords:** Cell Biology, Functional Aspects of Cell Biology

## Abstract

The ability of propionate, a short-chain fatty acid produced from the fermentation of non-digestible carbohydrates in the colon, to stimulate the release of anorectic gut hormones, such as glucagon like peptide-1 (GLP-1), is an attractive approach to enhance appetite regulation, weight management, and glycemic control. Propionate induces GLP-1 release via its G protein-coupled receptor (GPCR), free fatty acid receptor 2 (FFA2), a GPCR that activates Gαi and Gαq/11. However, how pleiotropic GPCR signaling mechanisms in the gut regulates appetite is poorly understood. Here, we identify propionate-mediated G protein signaling is spatially directed within the cell whereby FFA2 is targeted to very early endosomes. Furthermore, propionate activates a Gαi/p38 signaling pathway, which requires receptor internalization and is essential for propionate-induced GLP-1 release in enteroendocrine cells and colonic crypts. Our study reveals that intestinal metabolites engage membrane trafficking pathways and that receptor internalization could orchestrate complex GPCR pathways within the gut.

## Highlights

• Propionate activates Gαi/o but no Gαq/11 signaling in STC-1 cells or colonic crypts
• Acute Gαi/o signaling via FFA2 requires endocytosis and mediates GLP-1 release
• FFA2 traffics to very early endosomes to control rapid recycling and Gαi/o signals
• Propionate-induced internalization of FFA2 drives p38 signaling and GLP-1 release

## Graphical Abstract

**Figure:**
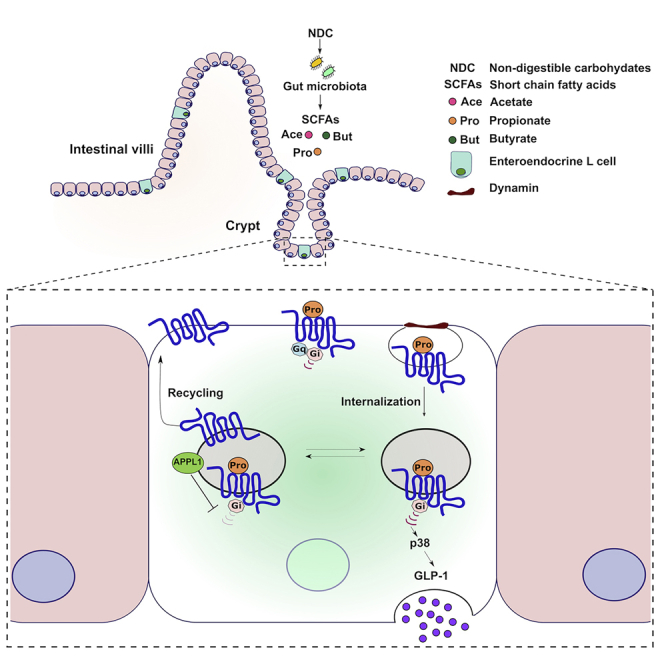

## Introduction

The consumption of dietary fiber, or non-digestible carbohydrates (NDCs), has been shown to protect against diet-induced obesity (Chambers et al., 2015). The protective effects of NDCs are largely attributed to short-chain fatty acids (SCFAs) that are produced in the colon by microbiota from the fermentation of NDCs (Chambers et al., 2015; Den Besten et al., 2013; James et al., 2003). Acetate, propionate, and butyrate are the predominant SCFAs produced and, in addition to regulation of gastro-intestinal functions, are involved in energy and glucose homeostasis and immune responses (Den Besten et al., 2013). Traditionally, roles of SCFAs in these metabolic processes were thought to be limited to their ability to act as an energy source or as a regulator of cholesterol synthesis; however, with the discovery and characterization of G protein-coupled receptors (GPCRs) activated by SCFAs, free fatty acid receptor 2 (FFA2, previously known as GPR43) and free fatty acid receptor 3 (FFA3, previously known as GPR41), it is now widely appreciated that many SCFA activities can be attributed to these receptors (Fuller et al., 2015; Li et al., 2018; Pingitore et al., 2019; Tolhurst et al., 2012; Bolognini et al., 2016).

Among the three SCFAs, propionate has been of particular translational interest owing to its ability to acutely suppress appetite via activation of FFA2 in enteroendocrine L cells and release of the anorectic gut hormones peptide YY (PYY) and incretin glucagon like peptide-1 (GLP-1) (Tolhurst et al., 2012; Psichas et al., 2015), contributing to its role in rapid weight loss and improved insulin sensitivity following Roux-en-Y gastric bypass (Liou et al., 2013). Direct health benefits of propionate in humans have been recently demonstrated whereby increasing the colonic levels of propionate in overweight humans not only exhibited reduced weight gain but also reduced abdominal adiposity and improved insulin sensitivity (Chambers et al., 2015). Thus propionate, and its receptor-mediated actions, represents an attractive system to develop therapeutic strategies in obesity management.

Although the role of SCFAs and their receptors in mediating the release of anorectic gut hormones has been demonstrated in rodent models and humans (Tolhurst et al., 2012; Bolognini et al., 2016; Psichas et al., 2015; Chambers et al., 2015), our understanding of the molecular mechanisms by propionate, regulating the release of anorectic gut hormone from enteroendocrine L cells, remains limited. FFA2 is coupled to both the Gαi/o and Gαq/11 families of heterotrimeric G proteins (Brown et al., 2003; Le Poul et al., 2003), although Gαq/11 is implicated in mediating gut hormone release via increases in calcium (Bolognini et al., 2016; Tolhurst et al., 2012). Models of GPCR signaling, however, have rapidly evolved over recent years from single receptors activating distinct G protein pathways at the plasma membrane, to high signal diversity that can be differentially activated by distinct ligands and exquisitely regulated at a spatial and temporal level. The spatiotemporal regulation of GPCRs can occur via a variety of processes, with membrane trafficking of GPCRs playing a central role. Membrane trafficking of GPCRs was classically viewed as a mechanism to control active cell surface receptor number by driving receptor internalization and post-endocytic sorting to divergent cellular fates. However, it is now understood that receptor internalization to endosomes provides additional intracellular signaling platforms including activation of heterotrimeric G protein signaling (Eichel and Von Zastrow, 2018; Hanyaloglu, 2018; Calebiro et al., 2009; Ferrandon et al., 2009). Endosomal signaling of GPCRs exhibits distinct functions from signaling activated at the plasma membrane, demonstrating the integrated nature of trafficking and signaling and providing a mechanism for cells to achieve highly specific and diverse downstream responses to its dynamic extracellular environment (Thomsen et al., 2018; Caengprasath and Hanyaloglu, 2019). Furthermore, we have previously shown that GPCRs are organized to distinct endosomal compartments to activate signaling (Sposini et al., 2017; Jean-Alphonse et al., 2014). These discoveries over the past decade have rewritten the GPCR “signaling atlas,” offering new interpretations of faulty GPCR activity in disease and providing novel therapeutic strategies to target GPCR signaling (Thomsen et al., 2018). However, the role of membrane trafficking for FFA2 and the distinct actions of propionate that activates pleiotropic G protein signal pathways within the gut remain unknown.

In this study, we demonstrate internalization-dependent FFA2 signaling drives propionate-induced GLP-1 release from enteroendocrine cells. Furthermore, we provide evidence that G protein signaling activated by FFA2 is differentially regulated by membrane trafficking and that an unexpected Gαi/p38 signaling pathway is required for propionate-induced GLP-1 release.

## Results

### Propionate Stimulates GLP-1 Secretion yet Activates Gαi/o but Not Gαq/11 Signaling from Colonic Crypts and Enteroendocrine Cells

Although propionate is known to mediate anorectic gut hormone release via FFA2, the ability of this SCFA to activate both upstream Gαq/11 and Gαi/o signal pathways in enteroendocrine cells has yet to be fully demonstrated. FFA2 couples to both Gαi/o to inhibit adenylate cyclase and reduce intracellular levels of cAMP and Gαq/11 that activates phospholipase C resulting in increases in inositol 1,4,5-triphosphate (IP_3_) and diacylglycerol, leading to mobilization of calcium from intracellular stores.

**Figure 1:**
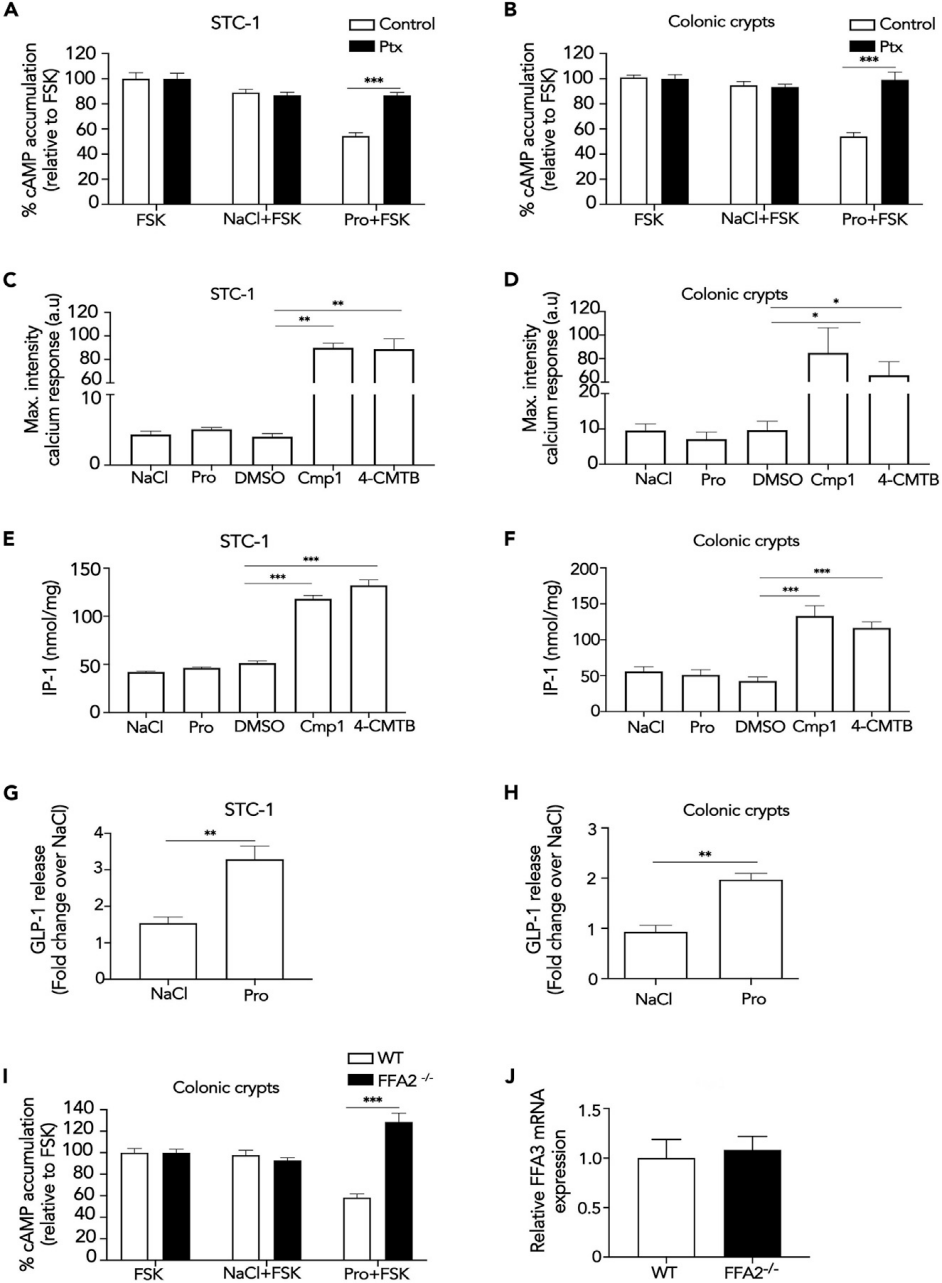
Propionate Stimulates GLP-1 Secretion and Activates Gαi/o but Not Gαq/11 via FFA2 (A and B) Intracellular cAMP levels measured in STC-1 cells (A) or colonic crypts (B) pre-treated with Pertussis toxin (Ptx; 200 ng/mL, 20 h) prior to pre-treatment with IBMX (0.5 mM, 5 min) and then stimulated with forskolin (FSK, 3 μM) or a combination of FSK with either NaCl or sodium propionate (Pro) (1 mM, 5 min). Data are expressed as percent change of FSK-treated cells. n = 3 independent experiments. Two-sided Mann-Whitney U test, ∗∗∗p < 0.001. (C and D) Intracellular calcium mobilization measured in STC-1 cells (C) or colonic crypts (D). Cultures were incubated with calcium indicator Fluo4-AM for 1 h and imaged live via confocal microscopy for 1 min before the addition of NaCl (1 mM), sodium propionate (Pro), DMSO, orthosteric FFA2 agonist Cmp1 (10 μM), or allosteric FFA2 agonist 4-CMTB (10 μM). Average maximal intensities of n = 20 cells in duplicate per six independent experiments. Two-sided Mann-Whitney U test, ∗p < 0.05. (E and F) Intracellular accumulation of IP_1_ in STC-1 cells (E) or colonic crypts (F). Cultures were treated with NaCl, sodium propionate (Pro) (1 mM), DMSO, Cmp1 (10 μM), or 4-CMTB (10 μM) for 2 h. STC-1 cells or crypts, n = 3 independent experiments. Two-sided Mann-Whitney U test, ∗∗∗p < 0.001. Data represent mean ± SEM. (G and H) (G) STC-1 cells and (H) colonic crypts were treated with either NaCl or sodium propionate (Pro) (1 mM, 2 h STC-1, 1 h crypts), and total GLP-1 levels secreted were measured via RIA. Data are expressed as fold change of total GLP-1 and normalized to basal (NaCl) secretion within the same experiment. For STC-1 cells, n = 4 independent experiments. For crypts, n = 3 independent experiments. Two-sided Mann-Whitney U test, ∗p < 0.05, ∗∗p < 0.01. (I) Intracellular cAMP levels measured in colonic crypts from wild-type (WT) or FFA2 knockout mice (FFA2 ^−/−^) pre-treated with IBMX (0.5 mM, 5 min) and then stimulated as in (C) and (D). Data are expressed as percent change of FSK-treated cells. n = 3 independent experiments. Two-sided Mann-Whitney U test, ∗∗∗p < 0.001. (J) Expression levels of FFA3 in WT and FFA2 ^−/−^ colonic crypts. mRNA isolated from colonic crypts of WT and FFA2 ^−/−^ mice were used in qPCR studies with specific mouse FFA3 primers. Data are presented as ΔΔCt. Two-sided Mann-Whitney U test. Data represent mean ± SEM. See also Figures S1–S4 and Videos S1, S2, and S3.

In both mouse enteroendocrine (STC-1) cells and colonic crypts, 1 mM propionate, a physiologically relevant dose that induced maximal responses in STC-1 cells and HEK 293 cells expressing FFA2 (Figures S1A and S1B) was able to inhibit forskolin-induced cAMP, which was significantly reversed by pre-treatment with Gαi/o inhibitor pertussis toxin (Ptx) (Figures 1A and 1B). Surprisingly, 1 mM propionate did not induce either an increase in intracellular calcium (Figures 1C and 1D and Video S1) or IP_1_, a downstream metabolite of IP_3_, in either STC-1 cells or colonic crypts (Figures 1E and 1F) despite its ability to induce GLP-1 release (Figures 1G and 1H). Treatment with a higher dose of propionate (10 mM) in both STC-1 cells and crypts also did not significantly increase intracellular IP_1_ or calcium levels (Figure S2). In contrast, a previously described selective FFA2 synthetic allosteric ligand (Lee et al., 2008), 4-CTMB, and a previously characterized selective FFA2 synthetic orthosteric ligand, compound 1 (Hudson et al., 2013) (Cmp1), activated both Gαi/o and Gαq/11 signaling in STC-1 cells and colonic crypts (Figures S1D and 1C–1F and Videos S2 and S3). Both compounds were also used at doses shown to induce maximal signal responses (Lee et al., 2008; Hudson et al., 2012 and Figure S1C), thus further demonstrating functional FFA2 in both cultures. The synthetic ligand-induced calcium responses were Gαq/11 mediated as they were significantly impaired by the pre-treatment of a selective Gαq/11 inhibitor, YM-254890 (Takasaki et al., 2004), in STC-1 cells (Figure S3).

Video S1. Propionate Is Unable to Increase Levels of Intracellular Calcium in Colonic Crypts, Related to Figure 1 Crypts were labeled with calcium indicator dye (Fluo4-AM) and intracellular calcium mobilization measured in live colonic crypts via confocal microscopy. Cultures were treated with sodium propionate (Pro) (1 mM).

Video S2. The Vehicle DMSO Is Unable to Increase Levels of Intracellular Calcium in Colonic Crypts, Related to Figure 1 Crypts were labeled with calcium indicator dye (Fluo4-AM), and intracellular calcium mobilization was measured in live colonic crypts via confocal microscopy. Cultures were treated with DMSO as a control for synthetic ligand Cmp1.

Video S3. An FFA2-Selective Synthetic Ligand Increases Levels of Intracellular Calcium in Colonic Crypts, Related to Figure 1 Crypts were labeled with calcium indicator dye (Fluo4-AM), and intracellular calcium mobilization was measured in live colonic crypts via confocal microscopy. Cultures were treated with Cmp1 (10 μM).

Despite the lack of detectable Gαq/11 signaling, propionate-induced-Gαi/o signaling was dependent on FFA2 as the reduction of forskolin-induced cAMP in colonic crypts derived from FFA2 knockout mice (FFA2 ^−/−^) was completely abolished (Figure 1I), consistent with our prior reports from the same mouse model that propionate-induced gut hormone release from the colon requires FFA2 (Psichas et al., 2015). This loss of propionate-mediated Gαi/o signaling in the FFA2 ^−/−^ crypts was not due to alterations in FFA3 expression (Figure 1J).

These data confirm that despite functional FFA2 responses, propionate activates Gαi/o signaling without detectable Gαq/11 responses in these cultures. To determine if the inability of propionate to activate Gαq/11 signaling via FFA2 was cellular context-specific, we stimulated HEK 293 cells expressing FLAG-FFA2. Treatment with 1 mM propionate significantly induced increases in intracellular calcium and IP_1_ (Figure S4) confirming activation of Gαq/11 signaling. Taken together, this demonstrates that, unlike synthetic FFA2 ligands, propionate is not able to signal via Gαq/11 in enteroendocrine cells, suggesting additional mechanisms beyond G protein activation are employed to induce propionate-mediated anorectic gut hormone secretion.

### FFA2/G Protein Signaling Is Spatially Regulated

**Figure 2:**
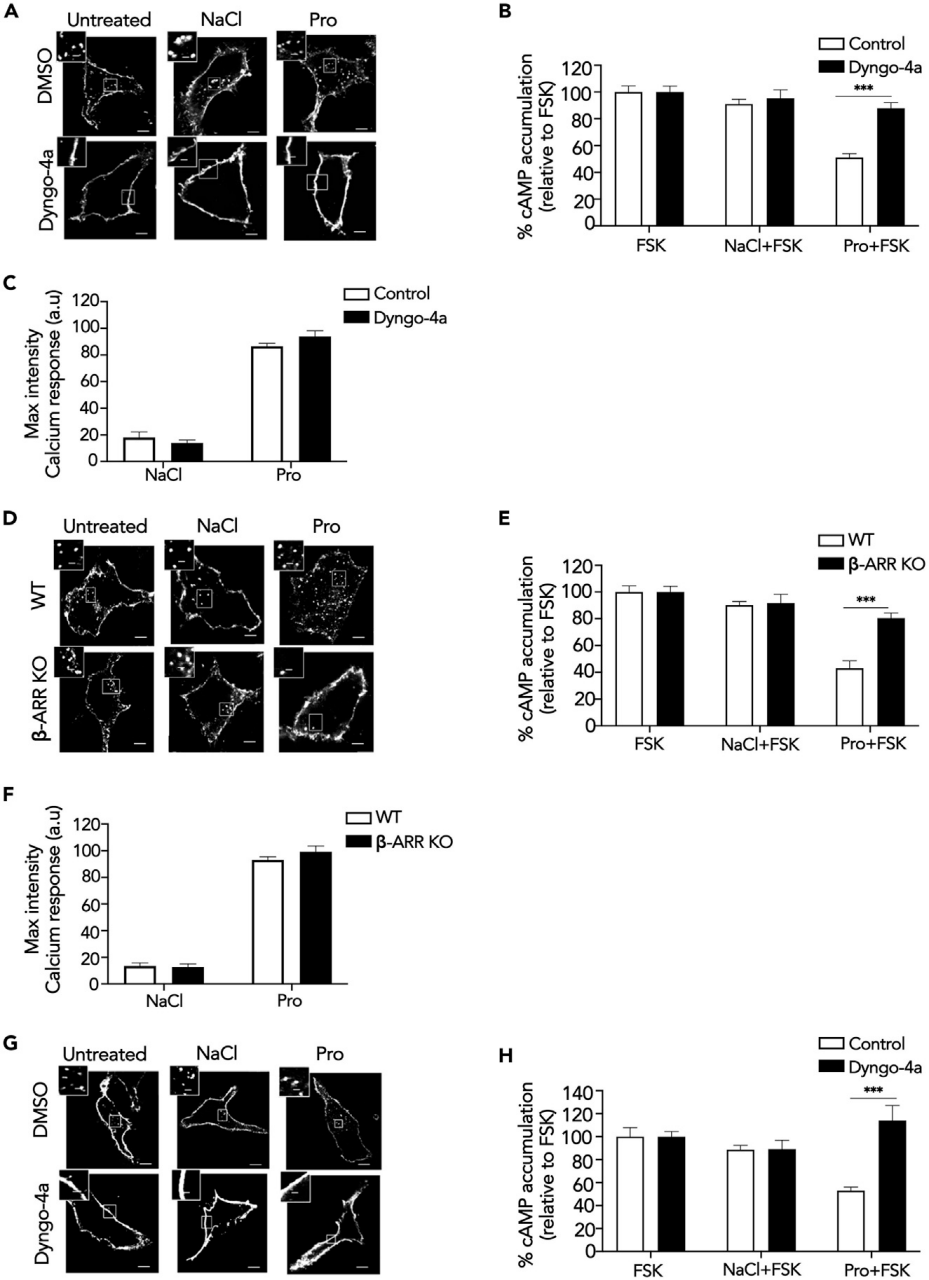
Propionate-Dependent Gαi/o Signaling Requires Receptor Internalization (A) Representative confocal microscopy images of HEK 293 cells expressing FLAG-FFA2 were pre-treated with either DMSO (vehicle) or Dyngo-4a (50 μM, 45 min), fed with M1 anti-FLAG antibody prior to stimulation with either NaCl or sodium propionate (Pro) (1 mM, 20 min). Fixed cells were imaged via confocal microscopy. (B and C) Intracellular cAMP levels (B) or calcium mobilization (C) measured in HEK 293 cells expressing FLAG-FFA2 pre-treated with either DMSO (vehicle) or Dyngo-4a (50 μM, 45 min). For (B), cells were pre-treated with IBMX (0.5 mM, 5 min) and then stimulated with forskolin (FSK, 3 μM) or a combination of FSK with either NaCl or sodium propionate (Pro) (1 mM, 5 min). n = 3 independent experiments. Two-sided Mann-Whitney U test, ∗∗∗p < 0.001. For (C), cells were incubated with calcium indicator Fluo4-AM for 1 h and imaged live via confocal microscopy for 1 min before the addition of either NaCl or sodium propionate (Pro) (1 mM). Average maximal intensities of n = 20 cells in duplicate per four independent experiments. (D) Representative confocal microscopy images of WT or β-ARR KO HEK 293 cells expressing FLAG-FFA2. Cells were treated with FLAG antibody and ligands and imaged as in (A). (E and F) Intracellular cAMP levels (E) or calcium mobilization (F) measured in WT or β-ARR KO HEK 293 cells transiently expressing FLAG-FFA2. Samples were treated and assayed as in (B) and (C). n = 3 independent experiments for either WT or β-ARR KO HEK 293 cells transiently expressing FLAG-FFA2. Two-sided Mann-Whitney U test, ∗∗∗p < 0.001. (G) Representative confocal images of STC-1 cells transiently expressing FLAG-FFA2 pre-treated with either DMSO (vehicle) or Dyngo-4a (50 μM, 45 min) then stimulated as in (A). (H) Intracellular cAMP levels of STC-1 pre-treated with either DMSO (vehicle) or Dyngo-4a (50 μM, 45 min). Scale bar, 5 μm; scale bar in inset, 1 μm. n = 3 independent experiments. Two-sided Mann-Whitney U test, ∗∗∗p < 0.001. For confocal images, representative images are shown of ~10 cells/experiment. Data represent mean ± SEM. See also Figure S5.

We next determined if propionate/FFA2 activation is spatially regulated via membrane trafficking as a potential mechanism underlying its actions in the gut. Many GPCRs undergo ligand-induced internalization via a well-described β-arrestin- and clathrin-dependent mechanism, whereby the large GTPase dynamin regulates the latter steps of endocytosis that drive clathrin-coated vesicle scission. To inhibit FFA2 internalization, the ability of a potent inhibitor of dynamin GTPase activity, Dyngo-4a, known to block the internalization of many GPCRs (Mccluskey et al., 2013; Eichel et al., 2016; Tsvetanova and Von Zastrow, 2014; Sposini et al., 2017), was first assessed in HEK 293 cells expressing FLAG-tagged FFA2 and imaged via confocal microscopy. Unexpectedly, FFA2 exhibited both constitutive and propionate-dependent internalization from the plasma membrane (Figure 2A), which was confirmed via flow cytometry (Figure S5A). In cells pre-treated with Dyngo-4a, a strong inhibition of both constitutive and propionate-induced FFA2 internalization was observed (Figure 2A), demonstrating that FFA2 constitutive and ligand-induced internalization occur in a dynamin-dependent manner. Under conditions where dynamin-dependent FFA2 internalization was inhibited in HEK 293 cells, the ability of propionate to inhibit forskolin-induced cAMP was impaired (Figure 2B). In contrast, FFA2-mediated Gαq/11 signaling, as measured by intracellular calcium responses (Figure 2C) or IP-1 accumulation (Figure S5B), was not significantly affected, suggesting a differential requirement of FFA2 internalization for FFA2-mediated signaling. These results were also confirmed in HEK 293 cells lacking β-arrestins 1 and 2 (Grundmann et al., 2018) (Figure S5C). Interestingly, only ligand-induced, but not constitutive, FFA2 internalization was inhibited by lack of β-arrestins (Figures 2D and S5D). However, as in cells pre-treated with Dyngo-4a, propionate-dependent inhibition of forskolin-induced cAMP was significantly impaired in β-arrestin knockout cells compared with wild-type HEK 293 cells (Figure 2E). In contrast, propionate-induced calcium mobilization and IP_1_ accumulation was unperturbed (Figures 2F and S5E).

The requirement of receptor internalization for Gα_i/o_ signaling was also determined for the endogenous propionate-responsive receptors expressed in STC-1 cells. As specific antibodies are not available for these receptors, STC-1 cells were transfected with FLAG-tagged FFA2 to confirm required conditions to inhibit FFA2 internalization in these cells. Similar to HEK 293 cells, FFA2 internalization exhibited both constitutive and ligand-induced endocytic profiles, and both were inhibited by Dyngo-4a (Figure 2G). Consistent with our observations in HEK 293 cells, Dyngo-4a pre-treatment inhibited propionate-mediated activation of Gαi/o signaling (Figure 2H). Overall, these data demonstrate a requirement for ligand-induced FFA2 internalization in propionate-mediated Gαi/o signaling in heterologous and enteroendocrine cells.

### FFA2 Internalizes to Very Early Endosomes for Sorting and Signaling

We have previously shown that GPCRs exhibit divergent sorting to distinct endosomal compartments between early endosomes (EEs) and very early endosomes (VEEs), and this post-endocytic organization is critical for both GPCR sorting fate and endosomal signaling (Jean-Alphonse et al., 2014; Sposini et al., 2017). As internalization of FFA2 is essential for its Gαi/o signaling, we next determined the postendocytic compartment that FFA2 internalizes to. The organization of FFA2 across VEEs and EEs was compared with the β2-adrenergic receptor (β2AR), a GPCR known to be rapidly sorted to the EE compartment (Jean-Alphonse et al., 2014). VEEs are one-third the diameter of EEs and lack classic EE markers such as early endosomal autoantigen 1 (EEA1); however, a subpopulation of VEEs contains the adaption protein APPL1 (adaptor protein phosphotyrosine interaction, pleckstrin homology domain, and leucine zipper containing 1), which plays essential roles in driving recycling from the VEE and in negative regulation of G protein signaling from this compartment (Jean-Alphonse et al., 2014; Sposini et al., 2017).

**Figure 3:**
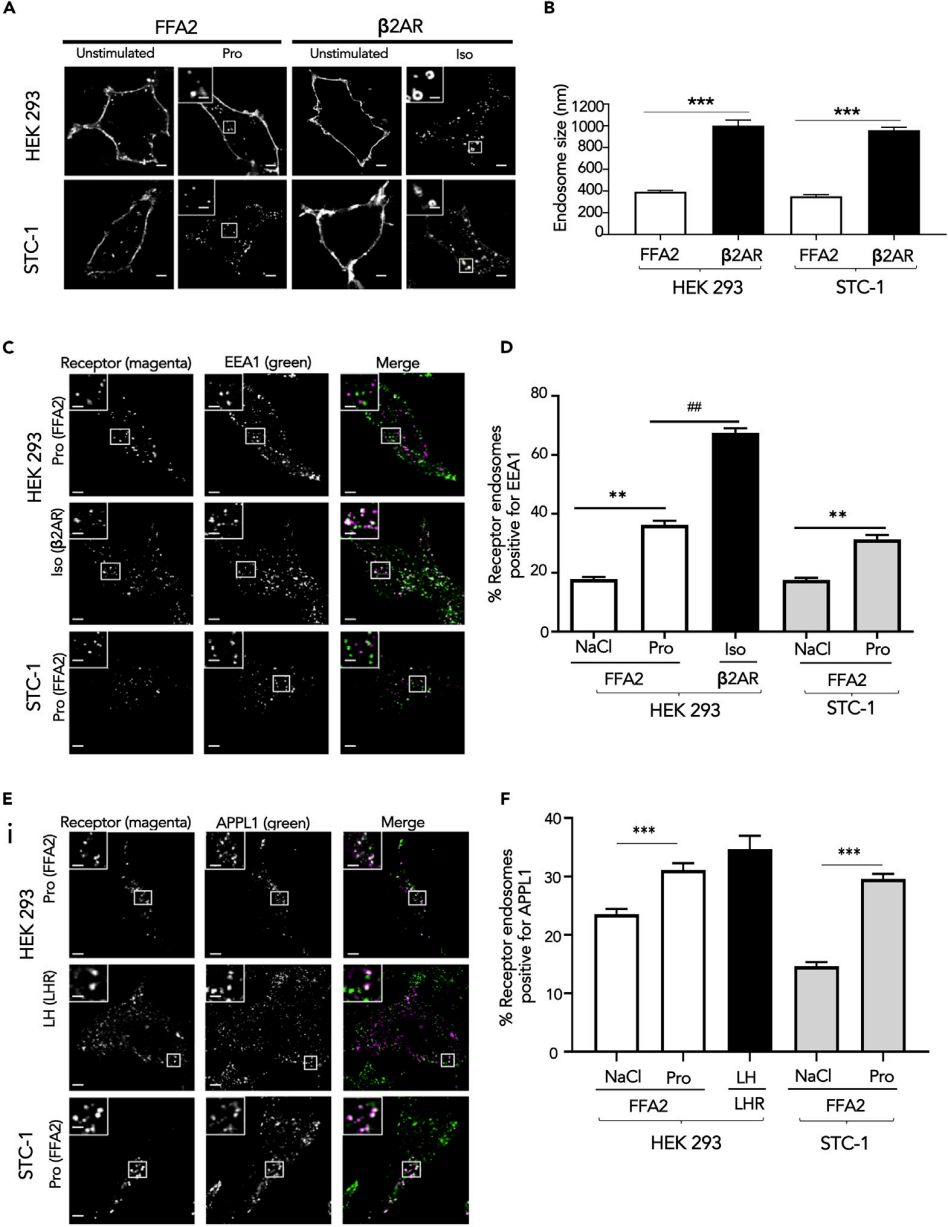
FFA2 Internalizes to Endosomes Exhibiting Properties of VEEs (A) Representative confocal microscopy images of HEK 293 cells expressing FLAG-FFA2 or FLAG- β2AR or STC-1 cells expressing FLAG-FFA2 or FLAG-β2AR imaged live with confocal microscopy before and after ligand treatment. FFA2 was stimulated with sodium propionate (Pro) (1 mM), and β2AR with isoproterenol (Iso, 10 μM) for 20 min. Scale bars, 5 μm; scale bar in inset, 1 μm. (B) Bar graph showing diameter of FFA2 or β2AR in HEK 293 or STC-1 cells containing endosomes. Endosome diameter was assessed by measuring the diameter of 20 endosomes, n = 10 cells per condition, collected across three independent experiments. Two-sided Mann-Whitney U test, ∗∗∗p < 0.001. (C) Representative confocal microscopy images of fixed HEK 293 cells stably expressing FLAG-FFA2 or β2AR or STC-1 cells transiently expressing FLAG-FFA2 treated with ligand for 20 min prior to “stripping” by PBS/EDTA (to remove surface bound FLAG antibody), fixation and stained with anti-EEA1 antibody. Scale bars, 5 μm; scale bar in inset, 1 μm. (D) Numbers of FFA2 or β2AR-containing endosomes positive for EEA1 quantified from (C); 200 endosomes per condition, 10 cells quantified per condition. Data represent mean ± SEM, n = 10 cells per condition, collected across three independent experiments. Two-sided Mann-Whitney U test, ∗∗p < 0.01, ##p < 0.01. (E, F) FFA2 colocalizes with APPL1. (E) Representative confocal microscopy images of fixed HEK 293 cells stably expressing FLAG-FFA2 or LHR or STC-1 cells transiently expressing FLAG-FFA2 treated with ligand (LH for LHR). Cells were treated as in (C) except that cells were stained with anti-APPL1 antibody. Scale bars, 5 μm; scale bar in inset, 1 μm. (F) Numbers of FFA2 or LHR-containing endosomes positive for APPL1 quantified from (E); 200 endosomes per condition, 10 cells quantified per condition. n = 10 cells per condition, collected across three independent experiments. Two-sided Mann-Whitney U test, ∗∗∗p < 0.001. For confocal images, representative images are shown of ~10 cells/experiment. Data represent mean ± SEM. See also Figure S6.

Internalization of FLAG-FFA2 was imaged in both live HEK 293 and STC-1 cells over time (0–30 min). FFA2 internalized to endosomes ∼400 nm diameter within 5 min of ligand stimulation (Figures 3A, 3B, and S6), in contrast to the significantly larger size of endosomes containing internalized β2AR (Jean-Alphonse et al., 2014) (Figure 3A). Furthermore, the majority (>60%) of constitutive and ligand-induced internalized FFA2 did not traffic to an EEA1-positive EE compartment, compared with ∼70% for β2AR that did localize to EEA1-positive endosomes (Figures 3C and 3D), suggesting that FFA2 may traffic primarily to VEEs than EEs. This was further supported by the finding that a subpopulation of internalized FFA2 co-localizes with APPL1 (32.8 ± 0.35% following propionate treatment), similar to that observed with the luteinizing hormone receptor (LHR; 35.2 ± 1.92%), a GPCR known to traffic to VEEs (Sposini et al., 2017) (Figures 3E and 3F).

**Figure 4:**
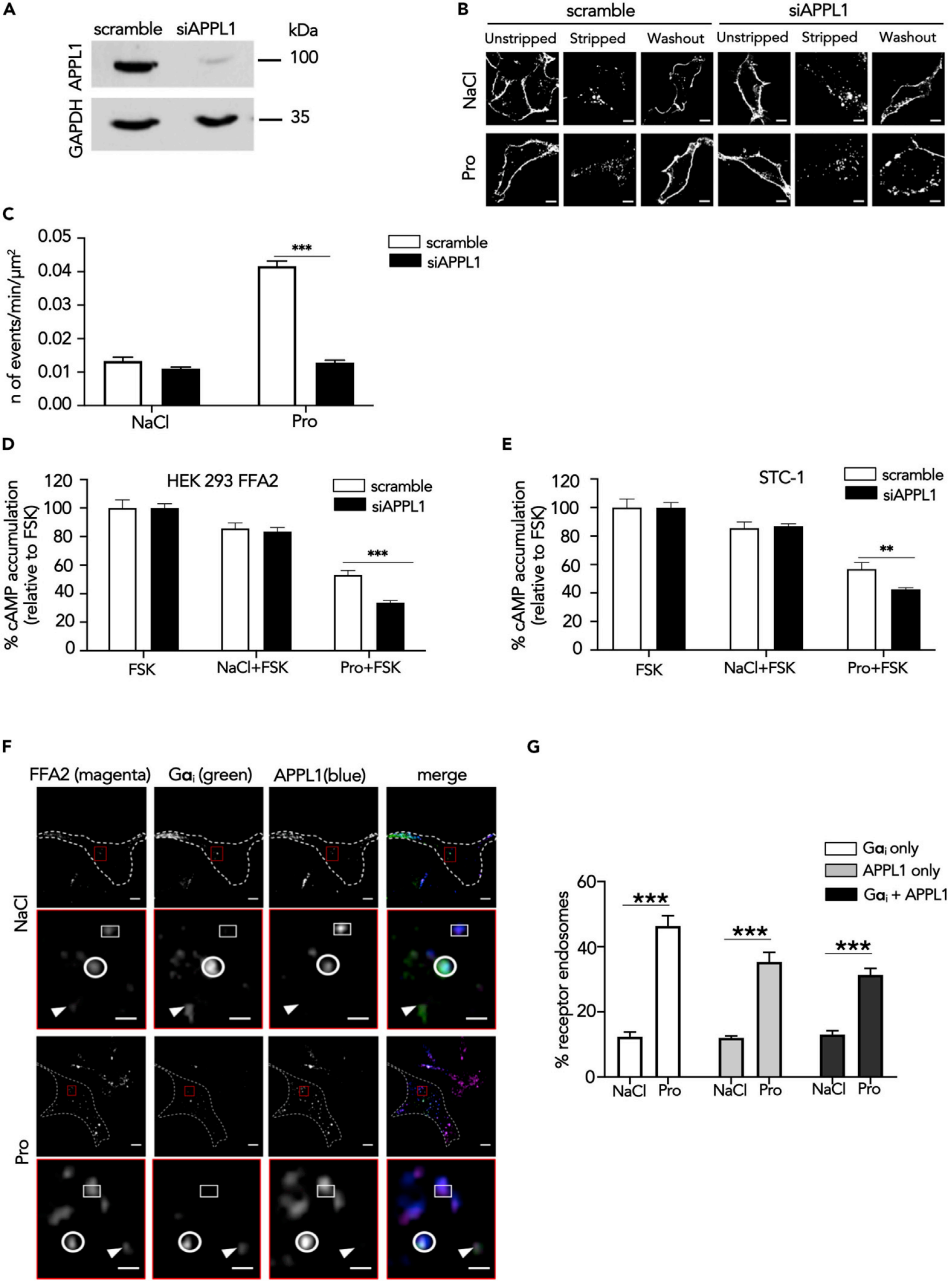
FFA2 Trafficking and G Protein Signaling Is Regulated by APPL1 (A) Representative western blot of total cellular levels of APPL1 from cells transfected with either scramble or APPL1 siRNA. GAPDH was used as a loading control. (B) Representative confocal microscopy images of propionate-induced internalization and recycling following APPL1 siRNA-mediated knockdown. HEK 293 cells stably expressing FLAG-FFA2 were labeled with anti-FLAG antibody and then treated with NaCl (1 mM) or propionate (Pro) (1 mM) for 20 min, then “stripped” and incubated with ligand-free medium for 1 h to allow receptor recycling. Scale bars, 5 μm. (C) Recycling of HEK 293 cells stably expressing SEP-FFA2 was measured in real time, via TIRFM; cells were transfected either with scramble or APPL1 siRNA and stimulated with NaCl (1 mM) or sodium propionate (Pro) (1 mM) for 5 min n = 20 cells per condition, collected across four independent experiments. Two-sided Mann-Whitney U test, ∗∗∗p < 0.001. (D and E) APPL1 negatively regulates propionate-mediated Gαi signaling. HEK 293 cells stably expressing FLAG-FFA2 (D) or STC-1 cells (E) transfected with either scramble of APPL1 siRNA prior to pre-treatment of IBMX (0.5 mM, 5 min) and then stimulated with forskolin (FSK, 3 μM) or a combination of FSK and NaCl or stated SCFAs (1 mM, 5 min). Data are expressed as percent change of FSK and NaCl treatment. n = 4 independent experiments. Two-sided Mann-Whitney U test, ∗p < 0.05; ∗∗p < 0.01. (F and G) FFA2 colocalizes with Gα_i_ within APPL1 endosomes. Representative TIRFM images of HEK 293 cells stably expressing FLAG-FFA2 (magenta), Gαi (green), APPL1 (blue) in cells stimulated either with NaCl (1 mM) or sodium propionate (Pro) (1 mM) for 5 min (F). Dotted line marks cell boundary. The lower panel of each treatment, highlighted in red, shows higher magnification image of the region of colocalization indicated by the red box in the corresponding upper-panel image. Arrows indicate FFA2 endosomes positive for Gαi only; circle indicates FFA2 endosomes positive for Gαi and APPL1; squares indicate FFA2 endosome positive for APPL1 only. Scale bars of upper-panel images, 10 μm; scale bar of lower-panel images 1 μm. (G) Quantification of FFA2 endosomes positive for Gαi, APPL1, or Gαi and APPL1; n = 12 cells per condition from (F) were quantified across three independent experiments. Two-way ANOVA, Bonferroni multiple comparisons test, ∗∗∗p < 0.001. Data represent mean ± SEM. See also Figures S7 and S8.

As FFA2 was primarily targeted to the VEE and propionate-induced FFA2 signaling requires internalization in enteroendocrine cells, we next investigated whether this compartment regulates FFA2 activity. We previously demonstrated that APPL1 is essential in rapid recycling of GPCRs targeted to this compartment back to the plasma membrane (Sposini et al., 2017). To determine a functional requirement of APPL1 on FFA2 trafficking, cellular levels of APPL1 were depleted via small interfering RNA (siRNA) in HEK 293 cells stably expressing FLAG-FFA2 (Figure 4A). We first examined the post-endocytic fate of FLAG-FFA2 when activated by propionate by confocal microscopy. APPL1 knockdown strongly impaired propionate-induced FFA2 recycling; in contrast, there was no effect in cells treated with NaCl (constitutive trafficking) as there was a complete return of the receptor back to the plasma membrane within 1 h following removal of ligand (Figure 4B). The role of APPL1 in rapid FFA2 recycling was quantitated via live-cell total internal reflection fluorescence microscopy (TIRFM) of an FFA2 tagged at the extracellular N terminus with pH-sensitive GFP super-ecliptic pHluorin (SEP). SEP fluoresces in an extracellular neutral pH environment but is non-fluorescent when confined to the acidic lumen of endosomes and therefore enables the detection of receptors upon insertion into the plasma membrane (Miesenbock et al., 1998; Yudowski et al., 2007). TIRFM imaging of SEP-tagged FFA2 (SEP-FFA2) established that FFA2 recycling events (identified as “puffs” of GFP fluorescence at the membrane [Figure S7A]) were transient and increased significantly within 5 min of propionate treatment, whereas treatment with NaCl exhibited a low rate of basal events (Figures S7B, S7C, and 4B), suggesting constitutively internalized FFA2 is sorted to a distinct slow recycling pathway. The frequency of these events remained constant throughout the duration of the imaging period (30 min) (Figure S7C). These plasma membrane insertion events were not affected by pre-treatment of cells with cycloheximide, suggesting that such events are not due to *de novo* receptor biogenesis (Figure S7D). In cells depleted of APPL1, however, propionate-induced, but not constitutive, recycling of SEP-FFA2 was significantly impaired (Figure 4C).

In addition to regulating the post-endocytic sorting of VEE targeted receptors, APPL1 also negatively regulates GPCR/Gαs signaling from this compartment (Sposini et al., 2017). As we have demonstrated that propionate-induced Gαi/o signaling requires receptor internalization, we next examined the potential role of the VEE in FFA2 signaling. Depletion of APPL1 resulted in a 2-fold increase in propionate-mediated inhibition of forskolin-stimulated cAMP (Figure 4D), suggesting that APPL1 can also negatively regulate heterotrimeric Gαi/o signaling in addition to Gαs signaling. Negative regulation of Gαi/o signaling by APPL1 was conserved in STC-1 cells as depletion of APPL1 levels also resulted in a significant enhancement of propionate-mediated inhibition of forskolin-induced cAMP (Figures S8 and 4E).

The above data suggest that propionate-induced Gαi/o signaling may occur from VEEs, and be regulated by APPL1 endosomes; therefore, we next determined whether FFA2 colocalizes with Gαi in APPL1-positive endosomes to determine if this G protein machinery is present in this compartment. HEK 293 cells expressing FLAG-FFA2 and Gαi-Venus were imaged via TIRFM as VEEs are prevalent in the peripheral juxtamembrane region of cells (Sposini et al., 2017). TIRFM analysis revealed that FFA2 and Gαi-Venus-positive endosomes are heterogeneous and characterized by FFA2-Gαi endosomes with and without APPL1. In addition, FFA2 endosomes were also positive for APPL1 where no Gαi-was present (Figures 4F and 4G). Overall, these data demonstrate that APPL1 is essential for propionate-induced FFA2 trafficking and regulation of propionate-mediated Gαi/o signaling.

### Propionate-Induced GLP-1 Release Requires Receptor Internalization and Gαi/o Signaling

**Figure 5:**
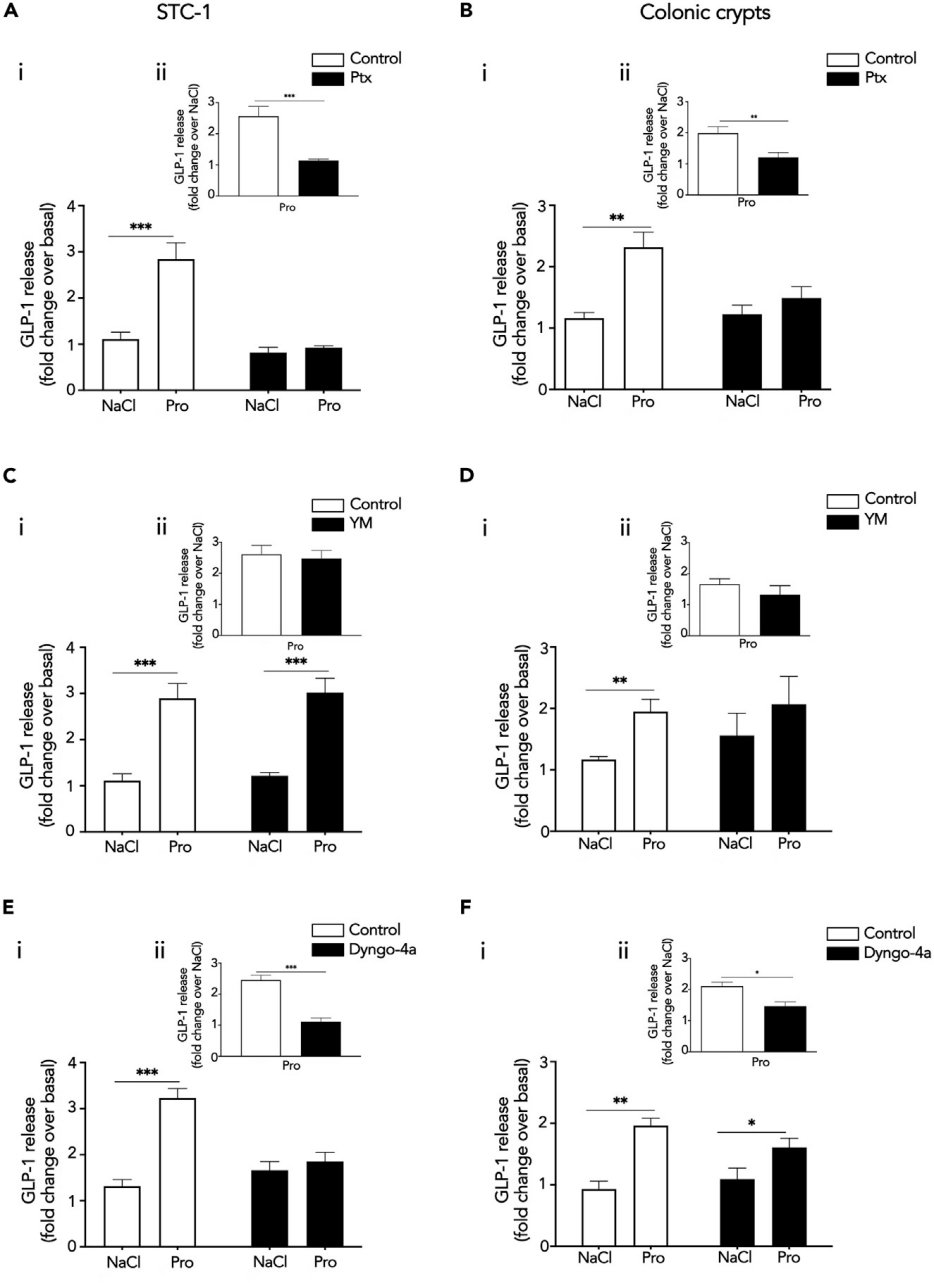
Endosomal Gαi/o Signaling Regulates Propionate-Mediated GLP-1 Release Stimulation of GLP-1 release from STC-1 cells (A) or colonic crypts (B) in the presence of Ptx. STC-1 cells or colonic crypts were pre-treated with either vehicle or Ptx (200 ng/mL, 20 h) prior to stimulation with either NaCl (1 mM) or sodium propionate (Pro) (1 mM) for 2 and 1 h for colonic crypts. For STC-1 cells, n = 4 independent experiments. For crypts, n = 8 independent experiments. Two-sided Mann-Whitney U test, ∗∗∗p < 0.001. Stimulation of GLP-1 release from STC-1 cells (C) or colonic crypts (D) in the presence of Gαq/11 inhibitor, YM-254890. STC-1 cells and colonic crypts were pre-treated with either DMSO or YM-254890 (YM, 10 μM, 5 min) and then treated as in (A) and (B). For STC-1 cells, n = 4 independent experiments. For crypts, n = 3 independent experiments. Two-sided Mann-Whitney U test, ∗p < 0.05, ∗∗p < 0.01. Stimulation of GLP-1 release from STC-1 cells (E) or colonic crypts (F) in the presence of Dygno-4a. STC-1 cells or colonic crypts were pre-treated with either DMSO or Dyngo-4a (50 μM, 45 min for STC-1 cells and 100 μM, 45 min for colonic crypts), following pre-treatment, Dyngo-4a was co-incubated with ligands for an additional 5 min and then removed. Cells and crypts were treated as in (A) and (B). For STC-1 cells, n = 5 independent experiments. For crypts, n = 3 independent experiments. Two-sided Mann-Whitney U test, ∗∗p < 0.01, ∗∗∗p < 0.001. Insets show propionate-induced GLP-1 release normalized to NaCl GLP-1 release. ∗∗p < 0.01, ∗∗∗p < 0.001. GLP-1 secretion of media and cells was detected via RIA and was expressed as fold change of total GLP-1 and normalized to NaCl secretion within the same experiment. Data represent mean ± SEM. See also Figures S9 and S10.

As propionate-induced Gαi/o signaling requires FFA2 internalization, we next assessed whether such a pathway regulates GLP-1 secretion. First, we examined the involvement of Gαi/o versus Gαq/11 signaling in mediating propionate-induced GLP-1 release, using Gαi/o or Gαq/11 inhibitors at concentrations that we demonstrated could inhibit receptor signaling in enteroendocrine cells (Figures 1A, 1B, and S3). STC-1 cells or colonic crypts pretreated with Ptx impaired propionate-induced GLP-1 release (Figures 5A and 5B). In contrast, pretreatment of STC-1 cells with the Gαq/11 inhibitor, YM-254890, had no significant effect on propionate-mediated GLP-1 release (Figure 5C). In colonic crypts, propionate-induced GLP-1 release in the presence of YM-25480 was impaired when assessing the ability of propionate to induce hormone release over NaCl treatment (Figure 5Di), although fold change responses were not significantly different between crypts treated with or without YB-254890 (Figure 5Dii). As we did not observe propionate-induced Gαq/11 signaling in colonic crypts (Figures 1F–1I), we determined if propionate-mediated FFA2 Gαi/o signaling is altered in the presence of YM-25480 in these primary cultures. In the presence of YM-25480, propionate-mediated inhibition of forskolin-induced cAMP was partially, but significantly, impaired compared with control treated cells (Figure S9A). This was only specific to propionate-mediated FFA2 signaling, as YM-25480 had no effect on propionate-mediated signaling from FFA3, a receptor coupled to Gαi/o and not Gαq/11 (Figure S9B). Together, these data suggest that, in STC-1 cells and colonic crypts, propionate induces GLP-1 secretion via a Gαi/o-dependent mechanism.

The requirement of Gαi/o signaling for propionate-mediated GLP-1 release, and the critical role of propionate-driven FFA2 internalization for G protein signaling, suggests a role for propionate-induced receptor internalization. To test this hypothesis, receptor endocytosis in STC-1 cells was blocked by Dyngo-4a treatment (Figure 2G). In cells treated with Dyngo-4a, propionate exhibited a marked reduction in GLP-1 release compared with control treated cells (Figure 5E). This inhibition by Dyngo-4a was not a result of an overall decreased capacity for these cells to secrete hormone as forskolin-induced GLP-1 release was not affected by inhibition of dynamin GTPase activity (Figure S10A). In colonic crypts, pretreatment with Dyngo-4a, impaired propionate-induced GLP-1 release but not to the same degree as observed in STC-1 cells (Figure 5F). As we have observed a dependence of propionate-driven FFA2 internalization for not only G protein signaling in STC-1 cells (Figure 2H) but also for GLP-1 release, we hypothesized that the differences in the inhibition of propionate-induced GLP-1 release by Dyngo-4a in colonic crypts may be due to more technical limitations of Dyngo-4a in primary crypts compared with a monolayer of cells. To assess this, we determined the ability of propionate to inhibit forskolin-induced cAMP in the presence of Dyngo-4a in colonic crypts. In the presence of Dyngo-4a, propionate-mediated inhibition of forskolin-induced cAMP was significantly, but only partially, impaired compared with control treated cells (Figure S10B), in contrast to the full inhibition of Gαi/o signaling by Dyngo-4a observed in STC-1 cells (Figure 2H). Thus, the level of propionate-dependent Gαi/o signal inhibition by Dyngo-4a correlates with its ability to inhibit propionate-driven gut hormone secretion. Overall, this suggests that internalization-dependent Gαi/o signaling mediates propionate-induced GLP-1 release.

### Internalization-Dependent Propionate Signaling Regulates GLP-1 Release via Activation of p38

**Figure 6:**
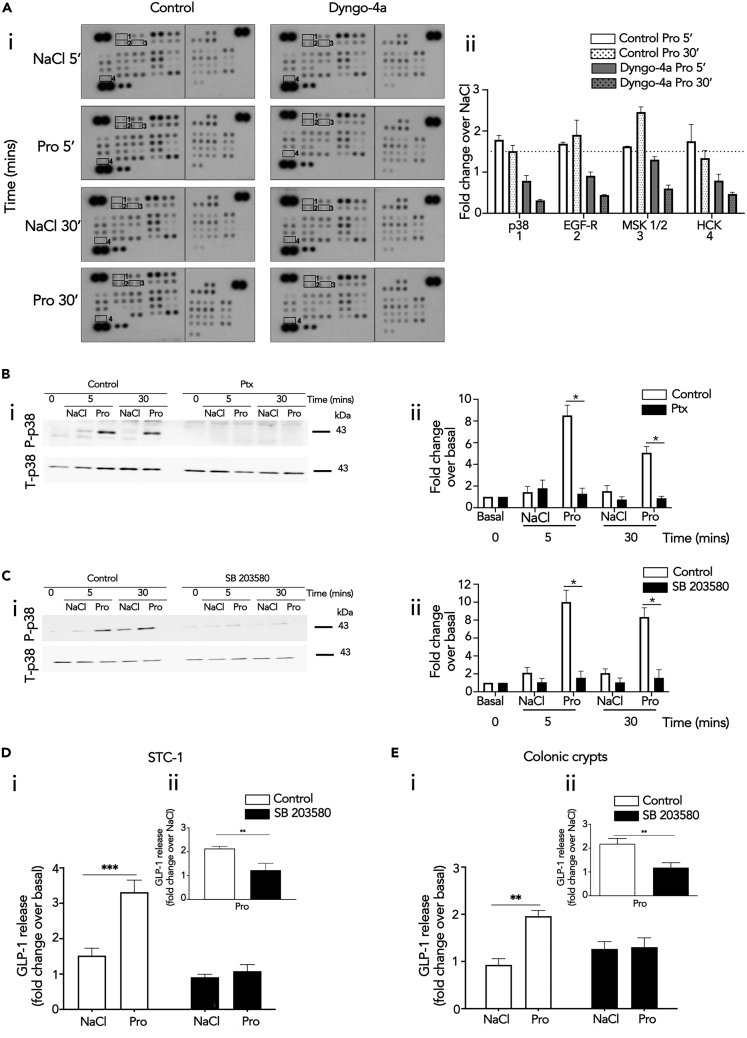
Endosomal Signaling of FFA2 Regulates GLP-1 Release via Activation of p38 (A) STC-1 cells were pre-treated with DMSO (vehicle) or Dyngo-4a (50 μM, 45 min) prior to stimulation with NaCl (1 mM) or propionate (Pro) (1 mM) for 5 or 30 min. Lysates were incubated with membranes spotted for 43 different phosphokinases (R&D systems). (Ai) Membranes highlighting location of phospho-kinase antibodies spotted onto the array. Signals of relevant kinases effected by Dyngo-4a are indicated by numbers. (Aii) Fold changes over NaCl in levels of phosphorylation that decreased in presence of Dyngo-4a. Data represent mean ± SEM of fold change values. (B–E) Representative western blots demonstrating phosphorylated p38 (P-p38) and total p38 (T-p38) of lysates from STC-1 cells pre-treated with either Ptx (B) or p38 inhibitor, SB 203580 (C). STC-1 cells were pre-treated with control or Ptx (200 ng/mL, 20 h) or SB 203580 (5 μM, 10 min) prior to stimulation of NaCl (1 mM) or propionate (Pro) (1 mM) at the indicated time points. Cell lysates were then collected for western blot analysis and probed for P-p38. Membranes were then stripped and re-probed with T-p38, which was used as a loading control (i). Densitometry and fold change analysis of P-p38 normalized to T-p38 of lysates pre-treated with Ptx, or SB 203580. Fold change of densitometry analysis of P-p38 levels normalized to basal of control or inhibitor at each time point stimulation with T-p38 (ii). Stimulation of GLP-1 release from STC-1 cells (D) or colonic crypts (E) in the presence of SB 203580. Both were pre-treated either with DMSO or SB 203580 (5 μM, 10 min), prior to stimulation with either NaCl (1 mM) or sodium propionate (Pro) (1 mM) for 2 h for STC-1 cells and 1 h for colonic crypts. For STC-1 cells and crypts, n = 3 independent experiments. Two-sided Mann-Whitney U test, ∗∗∗p < 0.001. Insets show propionate-induced GLP-1 release normalized to NaCl-induced GLP-1 release. ∗∗p < 0.01, ∗∗∗p < 0.001. GLP-1 secretion of media and cells detected via RIA and was expressed as fold change of total GLP-1 and normalized to NaCl secretion within the same experiment. Data represent mean ± SEM.

Increases in intracellular cAMP is an established driver of gut hormone release, yet our data indicates propionate induces gut hormone release in a Gαi/o-dependent manner, a pathway that decreases cAMP levels. Therefore, we hypothesized that the mechanism mediating endosomal Gαi/o-dependent GLP-1 release is potentially via distinct downstream pathways activated by Gαi/o, rather than its actions on its effector enzyme adenylate cyclase. Thus, we determined which propionate-mediated signaling pathways downstream of G protein signaling are also spatially regulated. A phosphokinase array was employed in STC-1 cells to identify propionate-induced signaling pathways dependent on receptor internalization. STC-1 cells were pretreated with Dyngo-4a and stimulated with propionate for 5 or 30 min. The array revealed that 16 of the 43 kinases within the array were phosphorylated after 5 or 30 min of propionate treatment. However, only p38α, EGF-R, MSK1/2, and Hck showed reduced phosphorylation when internalization was inhibited (Figure 6A). Of these kinases, p38α was selected for further analysis as this kinase and MSK1/2 are part of the same signal cascade. Furthermore, p38α is known to be activated at endosomes by other GPCRs (Grimsey et al., 2015). We then asked if propionate-induced p38 activation was Gαi/o-mediated. To test this, propionate-induced p38 activation was assessed in STC-1 pretreated with Ptx via western blot. Pretreatment of Ptx significantly impaired propionate-induced p38 signaling (Figure 6B).

Since propionate-induced activation of p38 involves receptor internalization and Gαi/o signaling, its role in propionate-induced GLP-1 release was assessed. A widely used selective p38 inhibitor SB 203580, which inhibits the catalytic activity of p38-α and -β isoforms without inhibiting p38 phosphorylation mediated by upstream kinases (Ge et al., 2002), was employed and significantly impaired propionate-induced activation of p38 (Figure 6C). In STC-1 cells and colonic crypts, SB 203580 pretreatment significantly impaired propionate's ability to induce GLP-1 secretion (Figures 6D and 6E) suggesting that propionate-induced Gαi signaling regulates GLP-1 release via a p38-dependent mechanism.

## Discussion

The ability of propionate to stimulate the release of anorectic gut hormones via the GPCR FFA2 represents a key physiological function of high interest owing to its demonstrated health benefits (Chambers et al., 2015, 2019). However, despite our increasing knowledge of the complexity of GPCR signaling networks in other cell systems, the underlying mechanisms regulating gut hormone release by propionate/FFA2 are poorly understood. In this study we demonstrate that signaling and downstream functions of FFA2, in response to propionate, are specified through tight control of receptor location.

In the gut, the current view is that GPCRs coupled to either Gαs-cAMP or Gαq/11- calcium pathways mediate anorectic gut hormone release (Hauge et al., 2017; Tian and Jin, 2016). From a receptor perspective, however, it is well known that many GPCRs are pleiotropically coupled, either directly or via receptor cross talk, and where additional mechanisms, such as intracellular receptor signaling, enable diversity in cell functions from the same G protein and second messenger system. Furthermore, different GPCR ligands (endogenous and synthetic) can elicit distinct conformational states and thus the potential to induce bias signal activity from the same receptor. In regard to FFA2 activity, which has been characterized previously as a dually coupled GPCR in studies primarily in heterologous cells (Brown et al., 2003; Le Poul et al., 2003), to date there have been no studies demonstrating its pleiotropic coupling to both Gαi/o and Gαq/11 in the gut at the level of second messenger signaling. Thus, to delineate the mechanisms of propionate-induced GLP-1 release from enteroendocrine cells, we first profiled the second messenger signaling activated by this SCFA in our intestinal models. Although propionate robustly signals via Gαi/o in a FFA2-dependent manner, it was unable to induce Gαq/11 signaling both in colonic crypts and STC-1 cells. This is in contrast to prior studies reporting a propionate-dependent calcium response in colonic cultures expressing Venus fluorescent protein in enteroendocrine L cells (Tolhurst et al., 2012). The reasons for this disparity are unclear but could relate to either the mouse model harboring Venus protein, longer culture times employed to create dispersed colonic cultures to measure calcium signaling, as opposed to the intact colonic crypts used in this study, and/or reflect a Gαi/o-mediated response as Gαi/o-coupled GPCRs are known to modulate calcium responses, including influx of extracellular calcium (Tang et al., 2015; Alkhatib et al., 1997). To our knowledge this is also the first demonstration that previously characterized synthetic orthosteric and allosteric FFA2 selective ligands activate Gαq/11 signaling in the colon. Although propionate may also activate FFA3, a Gαi/o-coupled receptor known to also be expressed in the colon, it was demonstrated in this study that colonic crypts from FFA2 KO animals are unable to activate SCFA-dependent Gαi/o signaling and that these animals do not exhibit altered FFA3 levels. This supports prior published work from us and others demonstrating SCFA-mediated GLP-1 release requires FFA2 (Tolhurst et al., 2012; Psichas et al., 2015). FFA2 is known to be a dually coupled receptor in HEK 293 or CHO cells (Le Poul et al., 2003), and indeed our data in HEK 293 cells expressing FFA2 are consistent with these reports, whereby propionate also activates Gαq/11 signaling. This potential system-dependent bias exhibited by FFA2 and propionate is intriguing given FFA2 can activate Gαq/11 signaling in enteroendocrine cells when stimulated with synthetic ligands. One potential mechanism for the distinct propionate/FFA2 signal profiles between enteroendocrine cells and heterologous cells is cross talk of FFA2 with another GPCR such as FFA3. However, it has recently been demonstrated that FFA2-Gαq/11 signaling is not decreased, but enhanced, via associations with FFA3 (Ang et al., 2018).

Propionate's inability to induce Gαq/11-mediated intracellular calcium mobilization is indeed paradoxical to what is known about the signaling requirements of anorectic gut hormone secretion (Spreckley and Murphy, 2015). Although we do not detect propionate-induced Gαq/11 signaling from FFA2, this does not exclude a role for calcium, from intracellular or extracellular sources, overall in downstream steps of GLP-1 secretion and modulation of the exocytic machinery. However, exocytosis is known to occur via either calcium-dependent or -independent pathways (Sato et al., 1998; Komatsu et al., 1995) and also involving Gαi (Aridor et al., 1993; Kreft et al., 1999; Mansvelder et al., 2002), including an ability for the Gαi-coupled FFA3 to induce GLP-1 release when stimulated with a FFA3-selective ligand (Nøhr et al., 2013). Although we observed that propionate-induced GLP-1 release was impaired in the presence of Ptx in STC-1 cells and colonic crypts, this is inconsistent with previous reports that did not find propionate-induced GLP-1 release to be modulated by Ptx (Tolhurst et al., 2012; Bolognini et al., 2016) but is impaired by the Gαq/11 inhibitor, FR900359 (Bolognini et al., 2016). However, confirmation of Ptx-dependent inhibition of propionate-mediated Gαi/o signaling at the second messenger level in STC-1 cells or crypts was not reported in these prior studies. Although FR900359 has been shown to also inhibit Gβγ-mediated signaling from Gαi/o-coupled receptors (Gao and Jacobson, 2016), we observed that inhibition of Gαq/11 activation partially inhibited propionate-FFA2 Gαi/o signaling but not FFA3 Gαi/o signaling, suggesting that an active Gαq/11 is integrated with FFA2 Gαi/o signaling. Such cross talk may be analogous to the findings that arrestin-mediated signaling of GPCRs requires an active G protein conformational state perhaps even in the absence of second messenger responses (Grundmann et al., 2018).

Our results demonstrating propionate-mediated GLP-1 release via Gαi/o suggest that mechanisms regulating propionate-induced release of GLP-1 may be more complex and not via Gαi/o-mediated decreases in cAMP levels per se, a second messenger that induces gut hormone release (Hauge et al., 2017). One mechanism that can diversify downstream cellular functions from a common upstream pathway is via spatial control of signaling. Indeed, agonist induced FFA2 internalization differentially regulated Gαi and Gαq/11 signaling, indicating at least in HEK 293 cells that FFA2/Gαi signaling was from internalized receptor and Gαq/11 signaling occurred from the plasma membrane. Spatial discrimination in GPCR/G protein signaling has been observed with the pleiotropically coupled calcium-sensing receptor (Gorvin et al., 2018). The specific requirement for receptor internalization in driving FFA2-mediated Gαi/o signaling was conserved in enteroendocrine cells, whereby this pathway mediated GLP-1 release, providing a novel mechanism underlying propionate's downstream functions in the gut. We also identified that FFA2 primarily traffics to the VEE in HEK 293 and STC-1 cells, an endosomal compartment we have previously shown to be critical for sorting and endosomal signaling for a subset of GPCRs (Jean-Alphonse et al., 2014; Sposini et al., 2017). For GPCRs that are trafficked to the small endosomes of the VEE, APPL1 has been demonstrated to be crucial for both receptor recycling and negative regulation of Gαs signaling. We demonstrate that rapid ligand-induced recycling of FFA2 is also APPL1 dependent and negatively regulates FFA2-endosomal Gαi/o signaling in HEK 293 and enteroendocrine cells, indicating that the APPL1/VEE compartment can negatively regulate distinct G protein pathways, in addition to Gαs-coupled GPCRs (Sposini et al., 2017).

Given the requirement for receptor internalization in Gαi/o signaling and propionate-induced gut hormone release, we hypothesized that additional Gαi/o-activated pathways were important in gut hormone secretion. We identified that phosphorylation of a small subset of downstream kinases required FFA2 internalization when activated by propionate, which suggests a potential role for endomembrane signaling in providing a signal platform to activate unique signaling substrates from the plasma membrane, or indeed other intracellular compartments (Eichel and Von Zastrow, 2018; Hanyaloglu, 2018). We focused on p38 as kinases of the same pathway, MSK1/2, were also identified in the array, and p38 is known to be activated at endosomes by other GPCRs (Grimsey et al., 2015). Propionate has also previously been shown to activate p38 in many cellular systems and has a role in regulating inflammatory responses (Rutting et al., 2019; Yonezawa et al., 2007; Ang et al., 2018). For enteroendocrine cells and colonic crypts, we identify a key role of p38 in regulating propionate-induced GLP-1 release. Interestingly, p38 is also involved in regulating GLP-1 secretion induced by meat hydrolysate and essential amino acid and low-molecular-weight chitosan (Reimer, 2006; Liu et al., 2013). More recently, propionate-induced GLP-1 release was also found to be regulated by p38 in chicken intestinal epithelial cells (Zhang et al., 2019), suggesting a conserved role of this kinase in anorectic gut hormone secretion induced by distinct metabolites. How p38 may regulate gut hormone secretion is unknown, although this kinase pathway may phosphorylate components of the SNARE complex such as syntaxin1a, shown recently to be essential for GLP-1 secretion in intestinal L-cells (Wheeler et al., 2017; Campbell et al., 2020).

Together, these findings support a model whereby propionate regulates anorectic gut hormone secretion by a tightly controlled mechanism involving integration of membrane trafficking and intracellular signaling. As more GPCRs are shown to traffic and be spatially regulated by this and other intracellular compartments, our studies may form the basis for a broader mechanism employed by intestinal metabolites, which activate multiple GPCRs within the gut, to diversify its functions *in vivo*.

### Limitations of the Study

This study demonstrates that the tight integral regulation of membrane trafficking and intracellular signaling is critical for propionate-induced secretion of anorectic gut hormone. However, a limitation of the current study is that it does not directly demonstrate that FFA2 signaling from the VEE specifically mediates gut hormone release. In addition to APPL1, other proteins that occupy the VEE are unknown. Furthermore, APPL1, although essential for regulating recycling and acute G protein signaling of GPCRs specifically targeted to the VEE, is also found in other compartments. Future studies will be directed to identify the proteins that comprise the VEE compartment and could be exploited to directly image and assess the role of signaling from this compartment in the intestine, and indeed other tissues where FFA2 is expressed. An additional consideration is that, although FFA2 trafficking is regulated in STC-1 cells, they are not polarized cells as in the intestinal crypt. Thus, post-endocytic organization of receptors across the VEE and other membrane trafficking compartments at the apical and basal side may represent an additional level of spatial organization.

### Resource Availability

#### Lead Contact

Further information and request for resources should be directed to and will be fulfilled by the Lead Contact, Aylin Hanyaloglu (a.hanyaloglu@imperial.ac.uk).

#### Materials Availability

All unique compounds and plasmids generated in this study are available upon request from the Lead Contact on a completed Materials Transfer Agreement.

#### Data and Code Availability

The datasets supporting the current study are available from the Lead Contact on request.

## Methods

All methods can be found in the accompanying Transparent Methods supplemental file.

## Supplemental Information

Document S1. Transparent Methods and Figures S1–S10
