# Supplementary material for: Internalization-Dependent Free Fatty Acid Receptor 2 Signaling Is Essential for Propionate-Induced Anorectic Gut Hormone Release
Source: iScience. 2020 Aug 12;23(9):101449. doi: 10.1016/j.isci.2020.101449 (PMC7452316; doi:10.1016/j.isci.2020.101449)
Supplement: Document S1. Transparent Methods and Figures S1–S10 [file mmc1.pdf]

iScience, Volume *23*

Supplemental Information

Internalization-Dependent Free Fatty Acid Receptor 2 Signaling Is Essential for Propionate-Induced Anorectic Gut Hormone Release

Natarin Caengprasath, Noemi Gonzalez-Abuin, Maria Shchepinova, Yue Ma, Asuka Inoue, Edward W. Tate, Gary Frost, and Aylin C. Hanyaloglu

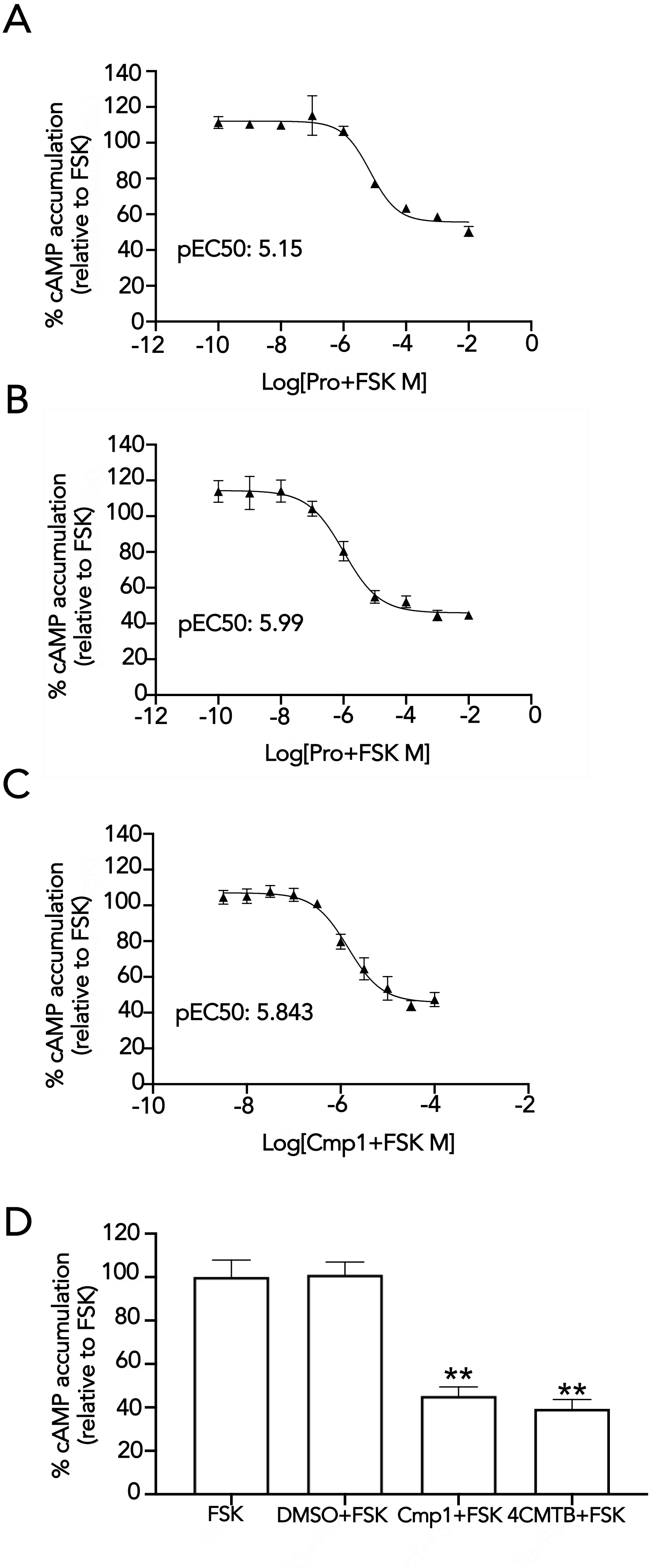

Figure S1 Propionate and selective FFA2 ligands activates Gαi/o signaling, related to Figure 1. (A-B) Dose-response curves for propionate induced inhibition of forskolin induced cAMP signalling in STC-1 cells (A) and HEK 293 cells expressing FLAG-FFA2 (B). (C) Dose-response curve for the synthetic FFA2-selective ligand Compound 1 (Cmp1) in Flp-In ™ T-Rex ™ HEK 293 cells expressing FFA2-eYFP. n = 3 independent experiments. Data points shown are the mean ± SEM. (D) Gαi/o signaling activated by FFA2 ligands was measured via inhibition of forskolin (FSK)-induced cAMP signaling in STC-1 cells. Cells were stimulated with either DMSO (untreated control), Compound 1 (Cmp1) or 4-CMTB (10uM, 5 min). n = 3 independent experiments. Two-sided Mann-Whitney U test, *** $p < 0.001$

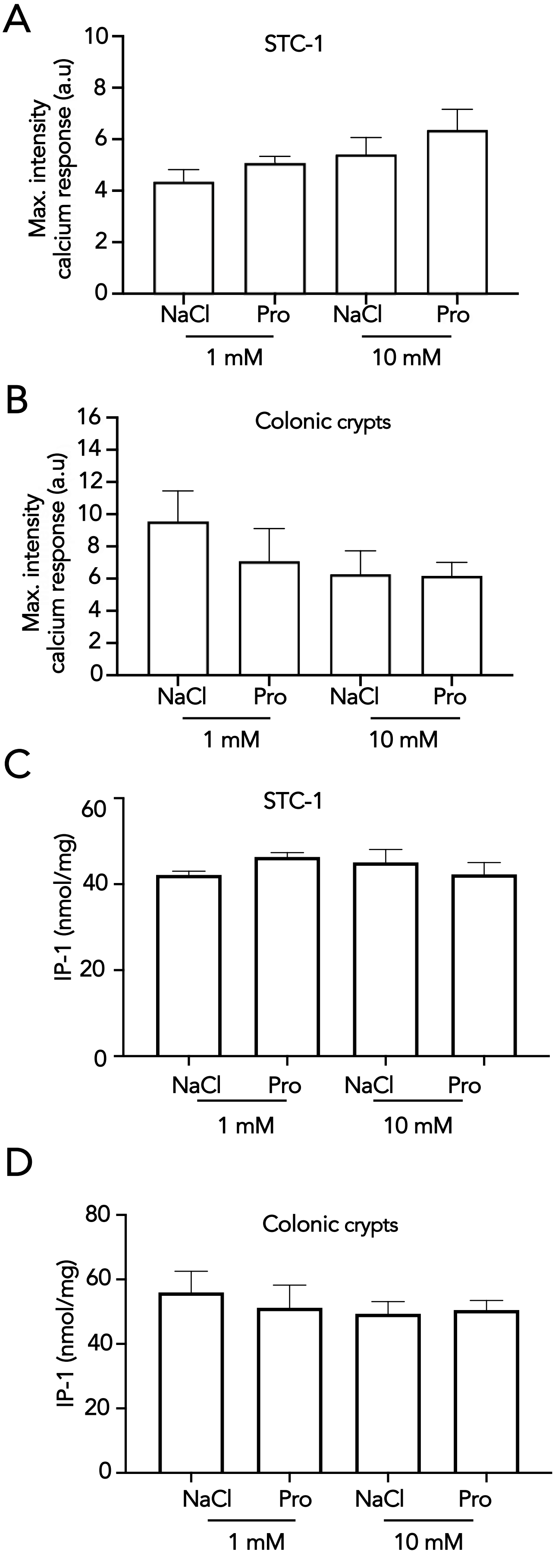

Figure S2 High concentrations of propionate are unable to signal via Gαq/11 in enteroendocrine cells, related to Figure 1. Intracellular calcium mobilization measured in STC-1 cells (A) or colonic crypts (B). Cultures were treated with either NaCl or sodium propionate (Pro) (1 and 10 mM). Average maximal intensities of n = 20 cells in duplicate per 5 independent experiments. Two-sided Mann-Whitney U test. Data represent mean ± SEM. Intracellular IP_1 levels measured in STC-1 cells (C) or colonic crypts (D). Cultures were treated with either NaCl sodium propionate (Pro) (1 and 10 mM). Two-sided Mann-Whitney U test. Data represent mean ± SEM.

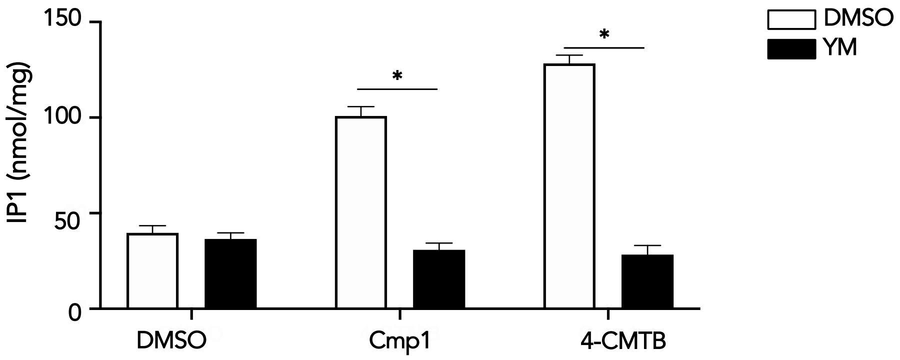

Figure S3 FFA2-mediated IP1 signaling requires active Gαq/11, related to Figure 1. Intracellular IP_1 levels measured in STC-1 cells, pre-treated with YM-254890 (YM, 10 μM, 5 min) and stimulated with either DMSO (untreated control), Compound 1 (Cmp1) or 4-CMTB (10 μM, 5 min). n = 4 independent experiments. Two-sided Mann-Whitney U test, *** p < 0.001.

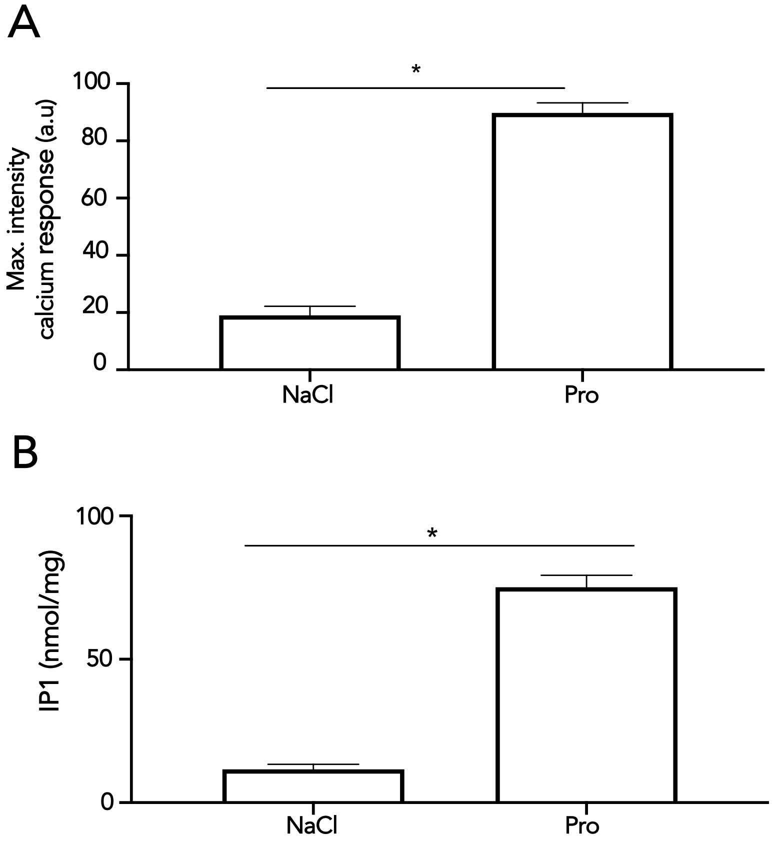

Figure S4. FFA2 actives both Gαi/o and Gαq/11 signaling in HEK 293 cells, related to Figure 1. Intracellular calcium mobilization (A) or intracellular accumulation of IP_1 (B) measured in HEK 293 cells expressing FLAG-FFA2. Cells were stimulated with either NaCl or sodium propionate (Pro) (1 mM). For intracellular calcium mobilization, average maximal intensities of n = 20 cells in duplicate per 4 independent experiments. Two-sided Mann-Whitney U test, * $p < 0.05$. For IP_1, n = 4 independent experiments. Two-sided Mann-Whitney U test, *** $p < 0.00$. Data represent mean ± SEM.

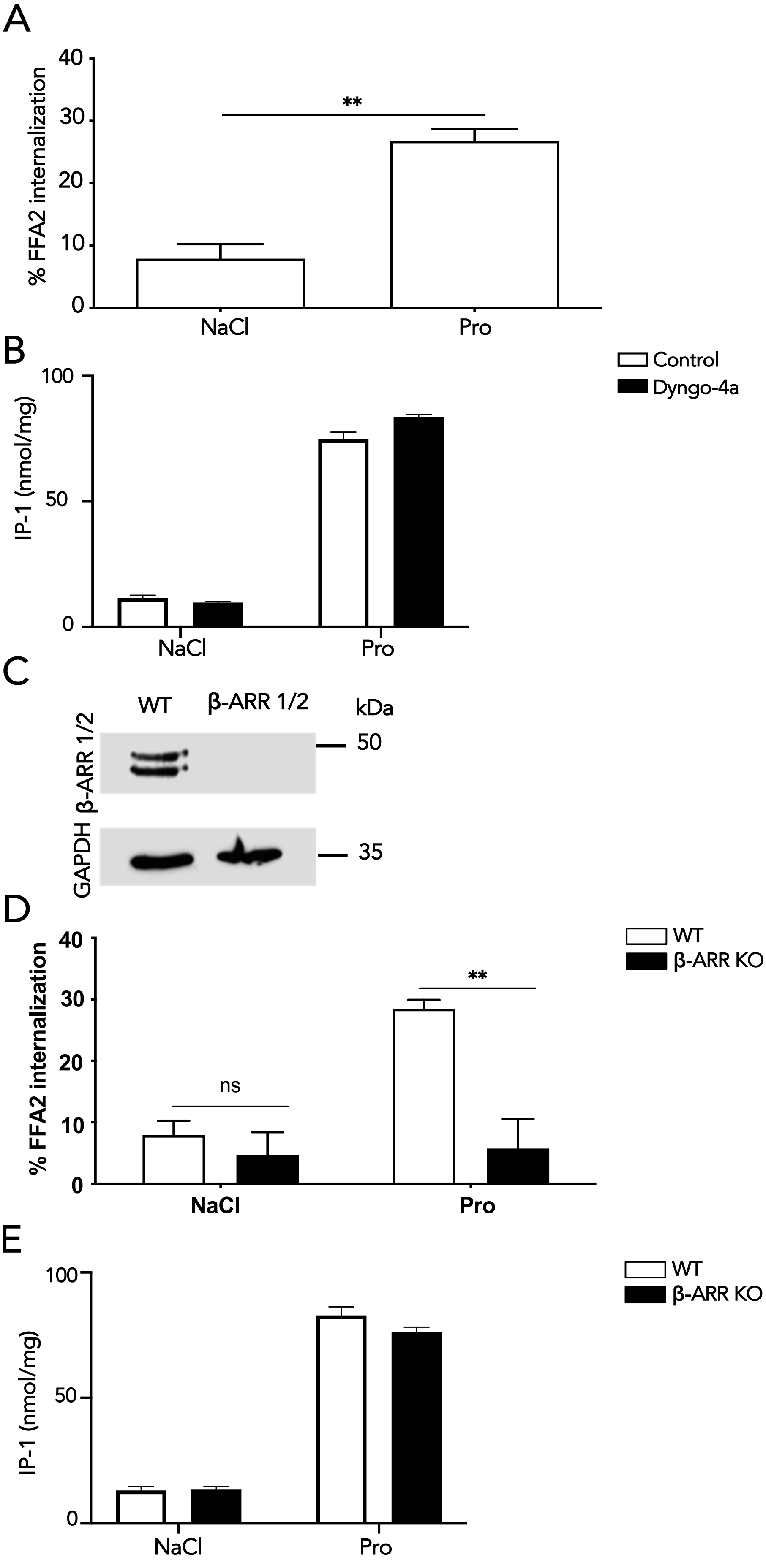

Figure S5 Differential spatial requirement for FFA2-mediated signaling, related to Figure 2. (A) FFA2 internalization measured by flow cytometry. HEK 293 cells transiently expressing FLAG-FFA2 were treated with and without NaCl (1 mM), or sodium propionate (Pro) (1 mM, 20 mins). FFA2 internalization was determined by decrease cell-surface labeling compared to untreated control. n = 4 independent experiments. Two-sided Mann-Whitney U test, ** $p < 0.01$. (B) Intracellular accumulation of IP_1 measured in HEK 293 cells transiently expressing FLAG-FFA2 pre-treated with either DMSO (vehicle) or Dyngo-4a (50 μM, 45 min) and treated with either NaCl or propionate (Pro) (1 mM, 2 h). n = 4 independent experiments. Two-sided Mann-Whitney U test. C) Western blot confirmation of the absence of β-arrestin 1/2

(β-ARR) expression in β-ARR KO HEK 293 cells compared to WT cells. GAPDH was used as loading control. (D) Dependence of FFA2 internalization on β-ARR measured by flow cytometry. WT and β-ARR KO HEK 293 cells transiently expressing FLAG-FFA2 were treated and data analysis were carried as in (A). n = 4 independent experiments. Two-sided Mann-Whitney U test, ** $p < 0.01$. (E) Intracellular accumulation of IP_1 measured in WT or β-ARR KO transiently expressing FLAG-FFA2 cells and treated as in (B). n = 24 independently plated wells for either WT or β-ARR KO transiently expressing FLAG-FFA2, representative of 4 independent experiments. Two-sided Mann-Whitney U test. Data represent mean ± SEM.

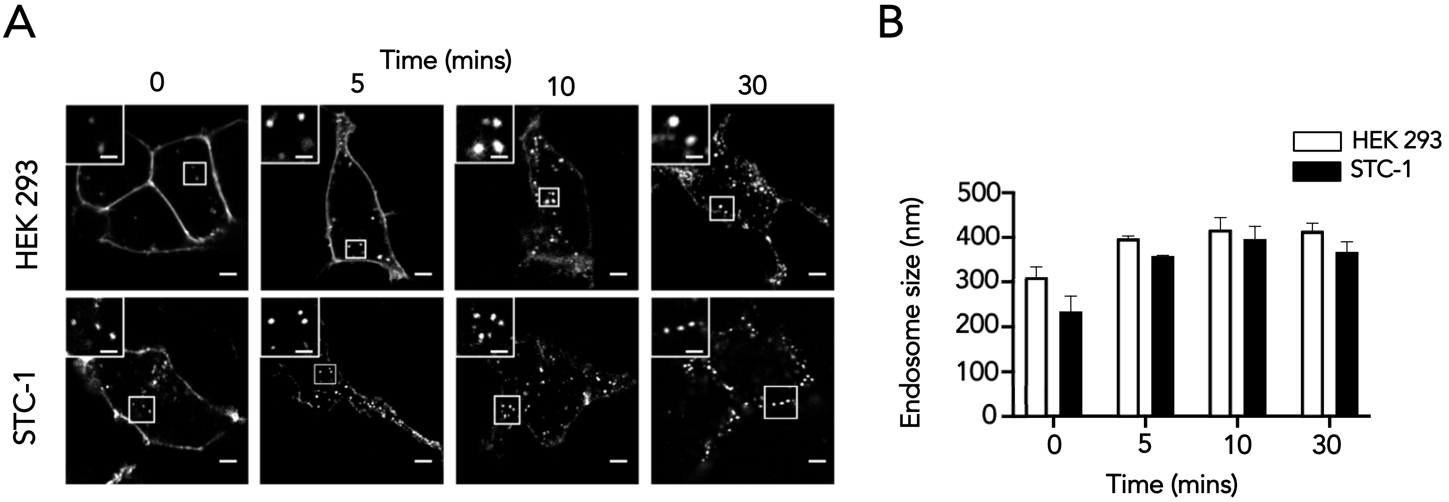

Figure S6 FFA2 internalizes to endosomes exhibiting properties of VEEs, related to Figure 3. (A) (i) Representative confocal microscopy images of STC-1 or HEK 293 cells transiently expressing FLAG-FFA2 imaged live via confocal microscopy before and after propionate treatment at different time points (1 mM). (ii) Bar graph showing diameter of FFA2 containing endosomes in HEK 293 or STC-1 cells. Endosome diameter was assessed by measuring the diameter of 20 endosomes, n = 10 cells per condition, collected across 3 independent experiments.

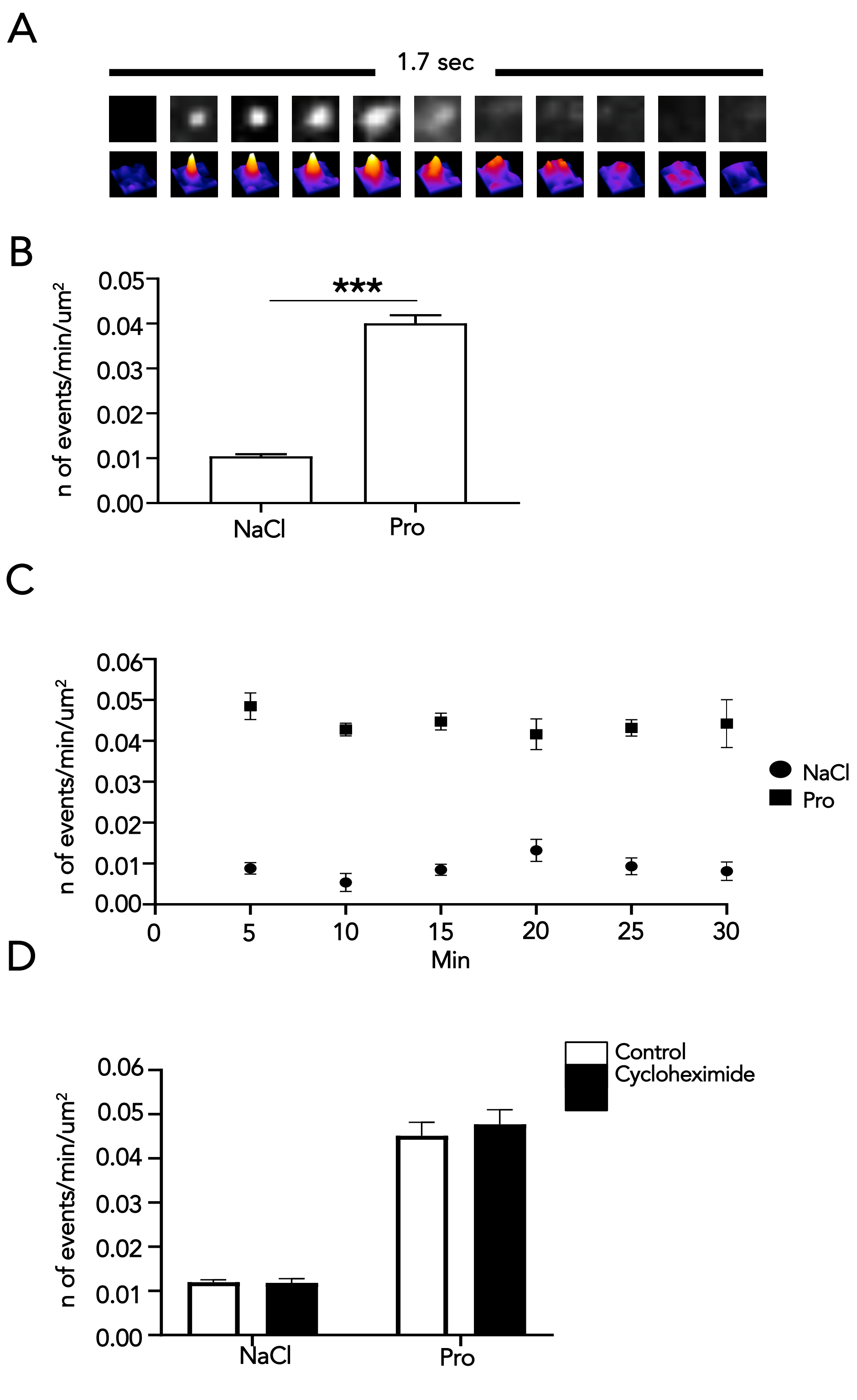

Figure S7 Characterization of SEP-FFA2 recycling events via TIRFM, related to Figure 4. (A) Series of TIRFM images of a single SEP-FFA2 recycling event following propionate stimulation with surface plot of fluorescence. (B) Number of recycling events measured by TIRFM in HEK 293 cells stably expressing SEP-FFA2 following stimulation of NaCl (1 mM) or propionate (Pro) (1 mM); n = 15 cells per condition; collected across 3 independent experiments. Two-sided Mann-Whitney U test, *** $p < 0.001$. (C) Number of recycling events over time following stimulation of NaCl (1 mM) or propionate (Pro) (1 mM) measured by TIRFM in HEK 293 cells stably expressing SEP-FFA2; n = 4 cells per time point; collected across 3 independent experiments. (D) Number of recycling events measured by TIRFM in HEK 293 cells stably expressing SEP-FFA2, pre-treated with DMSO (vehicle) or cycloheximide (10 µg/mL, 90 minutes) prior to NaCl (1 mM) or propionate (Pro) (1 mM) stimulation. n = 10 cells per condition, collected across 3 independent experiments. Two-sided Mann-Whitney U test. Data represent mean ± SEM.

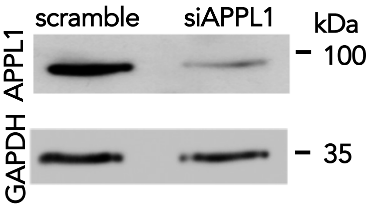

Figure S8 APPL1 knockdown in STC-1 cells via siRNA, related to Figure 4. Representative western blot of total cellular levels of APPL1 in lysates collected from STC-1 following either scramble (control), APPL1 (siAPPL1) siRNA-mediated knockdown. GAPDH was used as loading control.

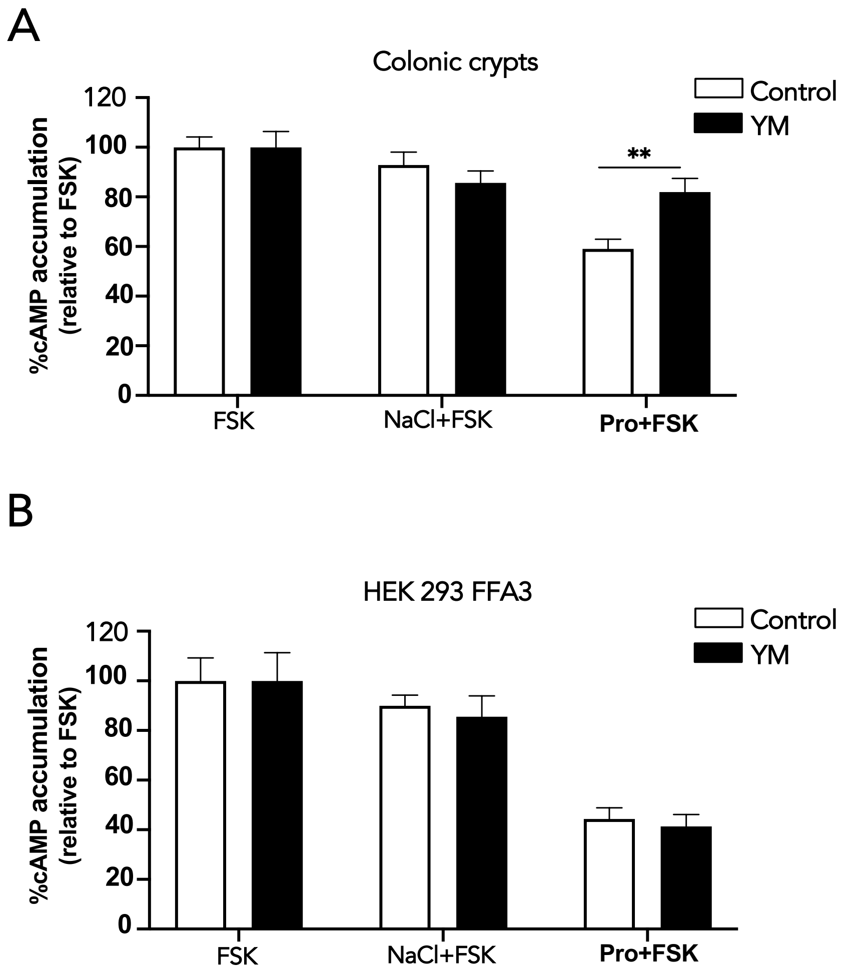

Figure S9 The Gαq/11 inhibitor, YM-254890, partially inhibits Gαi/o signaling activated by propionate/FFA2, related to Figure 5. (A) Gαi/o signaling activated by propionate (Pro) (1 mM, 5 min) in colonic crypts was measured via inhibition of forskolin (FSK)-induced cAMP signaling and pre-treated with or without YM-254890 (YM, 5 min, 10 mM). n = 4 independent experiments. Two-sided Mann-Whitney U test, **, $p < 0.01$. (B) HEK 293 cells transiently expressing FFA3, was pre-treated with and without YM, and Gαi/o signaling measured as in (A). n = 4 independent experiments. Two-sided Mann-Whitney U test, **, $p < 0.01$. Data represent mean ± SEM.

A

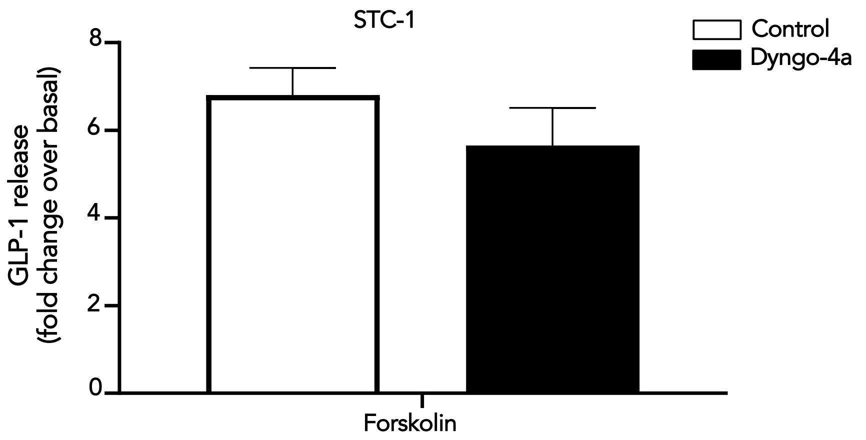

B

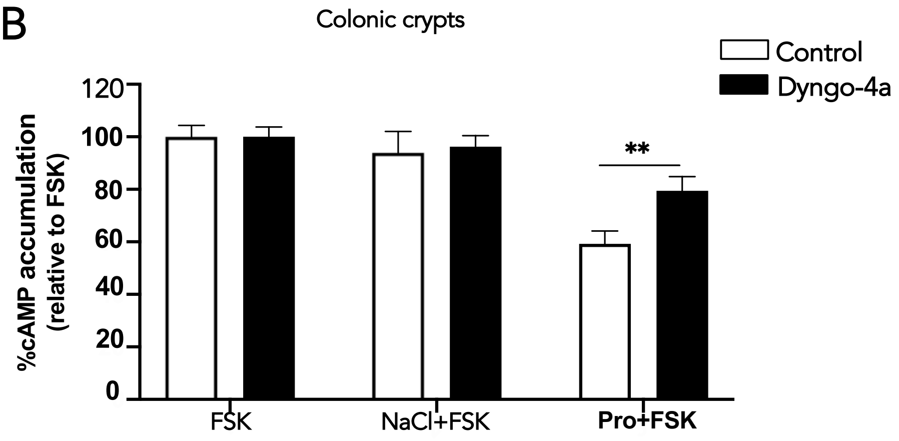

Figure S10 Dyngo-4 partially inhibits Gαi/o signaling activated by propionate/FFA2 in colonic crypts, related to Figure 5. (A) Forskolin induced GLP-1 release from STC-1 cells in the presence of Dygno-4a. STC-1 cells were pre-treated with either DMSO or Dyngo-4a (50 μM, 45 min). n = 5 independent experiments. Two-sided Mann-Whitney U test. (B) Gαi/o signaling activated by propionate (Pro) (1 mM, 5 min) in colonic crypts was measured via inhibition of forskolin (FSK)-induced cAMP signaling and pre-treated with or without Dygno-4a (50 μM, 45 min). n = 4 independent experiments. Two-sided Mann-Whitney U test, **, p < 0.01. Data represent mean ± SEM.

Transparent methods

Animals

C57BL/6J male mice purchased from Charles River were used to prepare mouse colonic crypts. FFA2 global knockout (FFA2 -/-) male mice were generated by Deltagen. FFA2 knockout was achieved by homologous recombination that replaces 55bp of FFA2 exon 1 with a cassette containing the neomycin resistance and β-galactosidase genes, resulting in a frameshift mutation (Maslowski et al., 2009). Animals were cared for in accordance with the British Home Office under UK Animal (Scientific Procedures) Act 1986 (Project License 00/6474).

Mouse colonic crypt culture preparation

Colons of wildtype (WT) or FFA2 -/- C57BL6 mice (8-12 weeks of age) were removed, cleaned and placed into ice-cold L-15 (Leibowitz) medium (Sigma). The intestinal tissue was thoroughly cleaned with L-15 medium and digested with 0.4 mg ml^{-1} collagenase (Sigma) in high-glucose DMEM at 37°C, as described previously (Psichas et al., 2015). The digestion process was repeated 4 times and resulting cell suspensions were centrifuged (5 min, 300 x *g*). The pellets were resuspended in DMEM (supplemented with 10% FBS and 1% antibiotics (100 U ml−1 penicillin and 0.1 mg ml^{-1} streptomycin)). Combined cell suspensions were filtered through a nylon mesh (pore size ~250 µm) and plated onto appropriate culture plates with wells coated with 2% Matrigel (Corning). The plates were incubated overnight at 37°C in an atmosphere of 95% O2 and 5% CO_2.

Colonic crypt FFA3 mRNA expression levels

Total RNA was extracted from plated WT and FFA2-/- (age-matched) colonic crypts using PureLink® RNA Mink Kit (Invitrogen) and DNase treated using on-column PureLink® DNase Treatment (Invitrogen). DNase-treated total RNA was reversed transcribed to a single-stranded cDNA using the high-capacity cDNA Reverse Transcription kit (Applied Biosystems). Quantitative reverse transcriptase PCR (qPCR) was carried out by QuantStudio® 12 K Flex Real-Time PCR System (Life Technologies) using TaqMan Gene Expression Assay (Applied Biosystems) with FFAR3 hydrolysis probe (Mm02621638_1, Applied Biosystems) and 18S as the reference gene (Eukaryotic 18S rRNA Endogenous Control, Applied Biosystems). The qPCR data are presented as relative expression levels calculated by ΔΔCt (where ΔCt is determined by the difference cycles threshold of the target gene and the reference gene).

Synthesis of Compound 1

All solvents and reagents were purchased from Sigma-Aldrich, Alfa Aesar unless otherwise stated, and used without further purification. Moisture sensitive reactions were performed in oven dried flasks, under a nitrogen atmosphere. Anhydrous solvents were dispensed using Pure Solv™ solvent drying towers (Innovative Technology Inc.) Analytical thin layer chromatography was carried out using Merck Si_{60}, F_{254} chromatography sheets. Spots were visualised by UV light or through use of an appropriate stain (ninhydrin or potassium permanganate). Flash column chromatography was run on a Biotage Isolera™ One flash purification system using a wet-loading Biotage SNAP cartridge. Mass spectra were acquired by the Imperial Mass Spectrometry service with *m/z* values reported in Daltons. ^1H spectra were recorded on a Bruker Av-400 (400 Hz) instrument at RT. Chemical shifts are expressed in parts per million δ relative to residual solvent as an internal reference. The multiplicity if each signal is indicated by: s = singlet; broad s= broad singlet; d = doublet; t = triplet; m= multiplet. Coupling constants (*J*), calculated using MestReNova© NMR software, are quoted in Hz and recorded to the nearest 0.1 Hz.

1 *N*-cyclopropyl-*N*'-benzoylthiourea

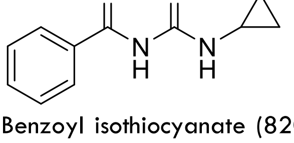

Benzoyl isothiocyanate (820 µL, 6.13 mmol, 1.0 eq.) was dissolved in CH_2Cl_2 (25 mL) at 0 °C, followed by a dropwise addition of cyclopropylamine (425 µL, 6.13 mmol, 1.0 eq.). The solution was then warmed up to RT and allowed to stir for 17h. The crude mixture was concentrated *in vacuo*, yielding benzoylthiourea as a yellow solid (1348 mg, quant.), which was used in the next step without further purification. ^1H NMR (400 MHz, $CDCl_3$): δ 10.93 (1H, broad t), 9.17 (1H, s), 7.83 (2H, dd, *J* = 8.4, 1.4 Hz), 7.59 (1H, t, *J* = 7.4 Hz), 7.48 (2H, t, *J* = 7.9 Hz), 3.23-3.17 (1H, m), 0.93-0.89 (2H, m), 0.80-0.77 (2H, m). The compound has been characterized in the literature, data in agreement (Olken and Marletta, 1992).

2 Cyclopropylthiourea

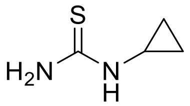

Benzoylthiourea **1** (900 mg, 4.09 mmol, 1 eq.) was dissolved in a solution of 5% (w/v) NaOH (20 mL) and heated to 80 °C. The solution was stirred for 3h and then cooled to RT in an ice/water bath. The reaction mixture was titrated to pH 8.0 with HCl_{conc}. The crude was extracted with EtOAc (4 x 15 mL). The organic fractions were combined, dried over anhydrous $MgSO_4$, filtered and concentrated *in vacuo*. The crude was redissolved in CH_2Cl_2 (10 mL) and precipitated with a dropwise addition of Et_2O to afford a thiourea **2** as a white-off solid (200 mg, 42%), which was used in the next step without further purification. ^{1}H NMR (400 MHz, DMSO-d_6): δ 2.35 (1H, broad s), 0.67-0.63 (2H, m), 0.49-0.44 (2H, m). The compound has been characterized in the literature, data in agreement (Olken and Marletta, 1992).

3 2-Bromo-1-(2,5-dichlorophenyl)ethanone

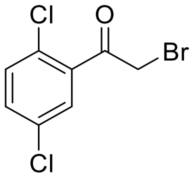

Dichloroacetophenone (230 µL, 1.59 mmol, 1.0 eq.) was dissolved in anhydrous MeCN (8 mL) and cooled to 0 °C under nitrogen, then NBS (312 mg, 1.75 mmol, 1.1. eq.) was added, followed by a dropwise addition of TMS·OTf (14 µL, 0.08 mmol, 0.05 eq.). The solution was warmed up to RT and allowed to stir for 17h under nitrogen. The reaction mixture was concentrated *in vacuo* and purified by column chromatography (1 to 5% EtOAc in Hexane over 10 CV), which afforded bromide as a white-off thick oil (235 mg, 55%, 80% pure by NMR). ^{1}H NMR (400 MHz, $CDCl_3$): δ 7.54 (1H, d, *J* = 2.4 Hz), 7.40 (1H, d, *J* = 2.3 Hz), 7.39 (1H, s), 4.49 (2H, s). The compound has been characterized in the literature, data in agreement (Roman et al., 2010).

4 *N*-cyclopropyl-4-(2,5-dichlorophenyl)thiazol-2-amine

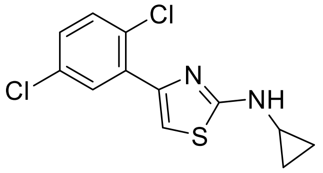

Cyclopropylthiourea **2** (50 mg, 0.43 mmol, 1.0 eq.) was dissolved in ethanol (2 mL), followed by addition of bromide **3** (80% pure, 138 mg, 0.52 mmol, 1.2 eq.) pre-dissolved in ethanol (1 mL). The solution was allowed to stir for 3h at RT and then concentrated *in vacuo*. The residue was dissolved in CH_2Cl_2 (5 mL), washed with saturated $NaHCO_3$ (4 mL), brine (4 mL). Organic layer was dried over $MgSO_4$, filtered and concentrated *in vacuo* to give a thick yellow oil. Column chromatography (1 to 10% EtOAc in Hexane over 10 CV) afforded amine **4** as an off-white thick oil (92 mg, 75%). R_f 0.57 (Hex:EtOAc = 3:1). ^{1}H NMR (400 MHz, $CDCl_3$): δ 7.86 (1H, d, *J* = 2.6 Hz), 7.37 (1H, d, *J* = 8.4 Hz), 7.19 (1H, dd, *J* = 8.3, 2.8 Hz), 7.13 (1H, broad s), 7.08 (1H, s), 2.60-2.54 (1H, m), 0.69-0.64 (2H, m), 0.56-0.52 (2H, m). The compound has been characterized in the literature, data in agreement (Hoveyda et al., 2018).

5 *tert*-Butyl (R)-3-benzyl-4-(cyclopropyl(4-(2,5-dichlorophenyl)thiazol-2-yl)amino)-4-oxobutanoate

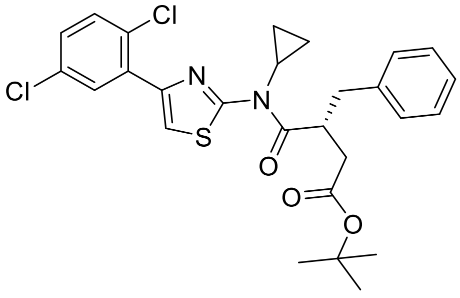

In a dry microwave vial (R)-2-benzyl-4-(tert-butoxy)-4-oxobutanoic acid (30 mg, 0.114 mmol, 1.3. eq.) was dissolved in anhydrous CH_2Cl_2 (1 mL) under nitrogen. Fluoro-*N*,*N*,*N*',*N*'-bis(tetramethylene)formamidinium hexafluorophosphate (BTFFH) (41 mg, 0.131 mmol, 1.5 eq.) was then added, followed by anhydrous i-Pr_2NEt (68 µL, 0.391 mmol, 4.5. eq.). The solution was allowed to stir for 30 min at RT under nitrogen, followed by addition of amine **4** (25 mg, 0.088 mmol, 1.0 eq.). The vial was then sealed, heated to 80 °C in an oil bath and allowed to stir for 18h. The yellow reaction mixture was cooled down to RT, further diluted with CH_2Cl_2 (5 mL), quenched with saturated NH_4CL (4 mL), washed with H_2O (4 mL) and brine (4 mL). The organic layer was dried over anhydrous

$MgSO_4$, filtered and concentrated *in vacuo* to give a dark-yellow thick oil. Column chromatography (1 to 10% EtOAc in Hexane over 10 CV) afforded amide **5** as a light-yellow thick oil (37 mg, 78%). R_f 0.65 (Hex:EtOAc = 3:1). ^{1}H NMR (400 MHz, $CDCl_3$): δ 7.99 (1H, d, *J* = 2.8 Hz), 7.62 (1H, s), 7.39 (1H, d, *J* = 8.6 Hz), 7.30-7.15 (6H, m), 4.23-4.13 (1H, m), 3.08 (1H, dd, *J* = 13.4, 6.7 Hz), 2.96-2.82 (2H, m), 2.73 (1H, dd, *J* = 13.4, 8.2 Hz), 2.44 (1H, dd, *J* = 16.4, 4.9 Hz), 1.39 (9H, s), 1.30-1.20 (3H, m), 0.85-0.78 (1H, m). LRMS (ES+): 531 ([$^{35}Cl^{35}ClM+H]^+$, 100%), 533 ([$^{35}Cl^{37}ClM+H]^+$, 75%), 535 ([$^{37}Cl^{37}ClM+H]^+$, 20%). The compound has been characterized in the literature, data in agreement (Hoveyda et al., 2018).

6 (R)-3-benzyl-4-(cyclopropyl(4-(2,5-dichlorophenyl)thiazol-2-yl)amino)-4-oxobutanoic acid (Cmp1)

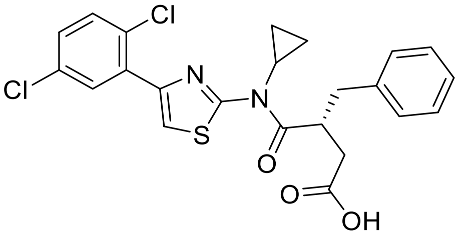

Amide **5** (10 mg, 0.019 mmol, 1.0 eq.) was dissolved in anhydrous CH_2Cl_2 (180 mL) under nitrogen, followed by addition of TFA_{conc} (40 mL, 20% (v/v)). The solution was allowed to stir for 4h at RT. The reaction mixture was concentrated *in vacuo*, redissolved in CH_2Cl_2 (3 mL) and quenched with saturated $NaHCO_3$ (3 mL). The organic layer was dried over anhydrous $MgSO_4$, filtered and concentrated *in vacuo*. The residue was redissolved in CH_2Cl_2 (3 mL) and precipitated with a dropwise addition of Et_2O to afford **Cmp1** as a beige powder (4.6 mg, 51%). The compound is unstable in solution under non-anhydrous conditions and decomposes to starting materials. The 10 mM stock of **Cmp1** in DMSO was immediately aliquoted and kept frozen at -20 °C. ^{1}H NMR (400 MHz, DMSO-d_6): δ 7.96 (1H, d, *J* = 2.7 Hz), 7.82 (1H, broad s), 7.61 (1H, d, *J* = 8.7 Hz), 7.47 (1H, dd, *J* = 8.4, 2.6 Hz), 7.32-7.14 (5H, m), 4.16-4.02 (1H, m), 3.07-2.81 (2H, m), 2.80-2.61 (2H, m), 2.38 (1H, d, *J* = 2.40 Hz), 1.28-1.16 (3H, m), 0.85-0.74 (1H, m).). LRMS (ES+): 475 ([$^{35}Cl^{35}ClM+H]^+$, 100%), 477 ([$^{35}Cl^{37}ClM+H]^+$, 75%), 479 ([$^{37}Cl^{37}ClM+H]^+$, 20%). The compound has been characterized in the literature, data in agreement (Hoveyda et al., 2018).

Plasmids

FLAG-FFA2 was generated by amplification of mouse FFA2 plasmid (MC203984, Origene) with forward 5'–GCTGATGACCCCAGACTGGCACAG–3' and reverse 5'–GCTCTAGACTACTCGGTGACAAATTCAGAACTCTG–3' primers and was ligated into XbaI and *AfeI* sites of the FLAG-LHR/pcDNA3.1 via digestion. SEP-FFA2 was generated by subcloning SEP from SEP-LHR using *XbaI* and *AfeI* sites and ligated to FFA2. FFA2-eYFP was generated by the amplification of mouse FFA2 (pFN21A Halo-mFFA2, Promega, UK) with forward 5'–*cagcctccggactctagcgtg*ccATGACCCCAGACTGGCAC–3' and reverse 5'–tgccgccgccgccCTCGGTGACAAATTCAGAACTC–3' primers that was fused with enhanced yellow fluorescent protein (eYFP) by the amplification of eYFP plasmid (expression vector pC1-mEYFP, in-house plasmid) with forward 5'–*tgtcaccgagggcggcggcggcagc*GTGAGCAAGGGCGAGGAGC–3' and reverse 5'–*tagaaggcacagtcgaggctcta*CTTGTACAGCTCGTCCATGC–3' primers. Amplified FFA2 and eYFP were inserted into linearized pcDNA5/FRT/TO plasmid (kindly provided by A. Tavassoli, G. Schmalzing and M. Rosenkilde labs) (forward 5'–*t*agAGCCTCGACTGTGCCTTC–3' and reverse 5'– ggcACGCTAGAGTCCGGAGGC –3' primers) using NEBuilder® HIFI DNA Assembly Master Mix (New England Biolabs). FLAG-LHR and SEP-LHR have been previously described (Jean-Alphonse et al., 2014; Sposini et al., 2017), respectively. FLAG- β2AR was a kind gift from Mark Von Zastrow, UCSF. Gαi-Venus was a kind gift from Johnathan Javitch, Columbia University. Gβ and Gγ were a kind gift from Eric Reiter, INRA, Nouzilly.

Cell Culture, transfections and stable cell lines

STC-1 (ATCC, male) and WT (ATCC) or β-ARR 1/2 KO (Asuka Inoue) HEK 293 cells (female) were maintained in high-glucose DMEM containing 10% FBS and penicillin/streptomycin (100 U/mL) at 37°C in 5% CO_2. Flp-In TREx HEK 293 cells (Francis Crick Institute) were maintained in high-glucose DMEM containing 10% FBS, 15 μg/mL blasticidin and 100 μg/mL zeocin at 37°C in 5% CO_2. For STC-1 or HEK 293 cells, transient transfections of plasmids were performed using Lipofectamine 2000 (Invitrogen) and cells were assayed 72 h post-transfection. Transfection of siRNA scramble or siRNA APPL1 (HSS119758, Life Technologies) was performed using RNAiMAX (Invitrogen) and cells were assayed 96 h post-transfection. To generate FLAG-FFA2 and SEP-FFA2 stable cell lines, FLAG- or SEP- FFA2 was transfected in HEK 293 cells using Lipofectamine 2000 with cells cultured in the presence of 0.5 μg/mL of geneticin. Geneticin-resistant clones were screen for receptor expression by flow cytometry and confocal microscopy. To generate Flp-In TREx HEK 293 cells able to inducibly express FFA2-eYFP, cells were cotransfected with pOG44 complementary vector and pcDNA5/FRT/TO-mFFA2-eYFP (9:1) using Lipofectamine 2000 with cells cultured in the presence of 100 μg/mL of hygromycin B to initiate selection of stably transfected cells. Following isolation of resistant cells, expression of FFA2-eYFP was induced by treatment with 1 μg/mL tetracycline one day prior to experiment.

Signaling assays
Intracellular cAMP was determined by homogenous time-resolved fluorescence (HTRF) (cAMP Dynamic 2 (CisBio) or LANCE® *Ultra* cAMP assay (PerkinElmer). In STC-1, HEK 293 cells or colonic crypts, cultures were pre-treated with IBMX (Sigma, 0.5 mM, 5 min) prior to ligand stimulation (5 min) and lysed with lysis buffer 4 (CisBio). The lysates were centrifuged at 12,000 x *g* for 10 mins and the supernatants were transferred to white 384-well plates. Anti-cAMP-Cryptate donor and cAMP-d2 acceptor were added to the wells and HTRF was measured using the PHERAstar (FSX) plate reader (BMG Labtech) equipped with 340 excitation filter and 620 nm (donor and 665 (acceptor) emission filter. For Flp-In TREx HEK 293 FFA2-eYFP cells, cells were detached, re-suspended in a freshly prepared stimulation buffer (5mM HEPES, 0.5 mM IBMX (Sigma), 0.1% BSA in Hank's Balanced Salt Solution at pH 7.4) and transferred to OptiPlate™-384 microplates (PerkinElmer) prior to ligand stimulation (30 min). EucAMP tracer and ULight-anti-cAMP solutions were added to the cells and incubated for 1h. Plates were read using an EnVision® Multimode Plate Reader (PerkinElmer) with a UV2 320 excitation filter and two emission filters (203 Eu 615 and 205 APC 665). Measurement of IP_1 was carried out using HTRF (IP-one (CisBio) by incubating cells or colonic crypts with ligands in serum free media supplemented with 50 mM LiCL (2h). Following stimulation, cultures were lysed with lysis buffer 4 (CisBio) supplemented with 50 mM LiCL and centrifuged at 12,000 x *g* for 10 mins and the supernatants were transferred to white 384-well plates. Anti-IP1-Cryptate donor and IP1-d2 acceptor were added to the wells and HTRF was measured using the PHERAstar (FSX) plate reader. cAMP and IP_1 concentrations determined by CisBio were corrected for protein levels. Calcium mobilization was measured by Fluo4-AM Direct Calcium Assay Kit (Invitrogen). Cells or colonic crypts were incubated with calcium dye in phenol red and serum free media for 30 min at 37°C and then at room temperature for 30 min. Cells or colonic crypts were imaged live using Leica SP5 confocal microscope using a 20X dry objective and a 488nm excitation laser. Movies were recorded at 1 frame per second for 1 min prior to ligand addition and a further 5-10 min following ligand addition to allow for calcium levels to lower to basal. The human phospho-kinase array kit (R&D system) containing 46 phosphokinases printed in duplication was used to identify a signaling pathway. STC-1 cells were serum-starved for 2 h prior to ligand stimulation and then lysed with array lysis buffer. Cell lysates were centrifuged at 12,000 x *g* for 10 min and protein concentrations were determined using Coomasie (Bradford) protein assay (Pierce). Protein lysates were incubated with the antibody-array membrane overnight and was then incubated with cocktail-detection antibody and streptavidin horseradish peroxidase. Protein signals were detected by the array Chemireagent and exposed to autoradiography film (GE healthcare). Spot intensity was quantified with ImageJ. For the measurement of p38 activation by western blot, STC-1 cells were serum-starved for 2 h prior to ligand stimulation. Following ligand stimulation, cells were rapidly washed with cold PBS and harvested with lysis buffer (1% Triton X-100, 50 mM Tris-HCl (pH 7.4), 150 mM NaCl, 0.5 mM EDTA, 1 mM NaF, 1 mM $NaVO_3$ and a protease inhibitor tablet (Roche)). Cell lysates were centrifuged at 12,000 x *g* for 10 min and protein concentrations were determined using Coomassie (Bradford) protein assay. Protein lysates were denatured at 95°C for 5 min in lysis buffer and Laemmli loading buffer containing 2% β-mercaptoethanol and separated on a 12% Tris-glycine polyacrylamide gel and transferred to nitrocellulose membranes. Membranes were incubated overnight with anti-rabbit phospho-p38 MAPK antibody (9211, Cell Signalling, 1/500) or anti-rabbit p38 MAPK antibody (9212, Cell Signalling, 1/1000) as a loading control. Specific protein bands were visualized using the ImageQuant Las 4000 chemi-image (GE Healthcare) and Luminata Forte HRP substrate (Millipore). Densitometry of the bands were quantified with ImageJ. Pre-treatments with either Ptx (Tocris), YM-254890 (Wako), Dyngo-4A (Abcam) or SB 203580 (Abcam) were carried out by incubating cells or colonic crypts for 20 h with 200 ng/mL Ptx, 5 min with 10 µM YM-254890, 45 min with 50 µM Dyngo-4a or 10 min with 5 µM SB 203580 before the addition of ligands. Experiments were conducted in duplicates for calcium mobilization and triplicates for all other experiments and were repeated at least three times.

Western blot
To determine total cellular levels of β-ARR 1/2 and APPL1, cells were lysed with lysis buffer (1% Triton X-100, 50 mM Tris-HCl (pH 7.4), 150 mM NaCl, 0.5 mM EDTA, 1 mM NaF, 1 mM $NaVO_3$ and a protease inhibitor tablet (Roche)). Cell lysates were centrifuged at 12,000 x *g* for 10 min and protein concentrations were determined using Coomassie (Bradford) protein assay. Protein lysates were denatured at 95°C for 5 min in lysis buffer and Laemmli loading buffer containing 2% β-mercaptoethanol and separated on a 12% Tris-glycine polyacrylamide gel and transferred to nitrocellulose membranes. Membranes were incubated overnight either with anti-rabbit β-ARR 1/2 (3858, Cell Signalling, 1/1000) or anti-rabbit APPL1 (3858, Cell Signalling,1/400) and then with anti-mouse GAPDH antibody (AB2302, 1/10,000, Millipore) as a loading control. Specific protein bands were visualized using the ImageQuant Las 4000 chemi-image (GE Healthcare) and Luminata Forte HRP substrate (Millipore).

GLP-1 secretion assays
Plated STC-1 cells and colonic crypts were washed with secretion buffer (HBSS supplemented 1% BSA fatty acid free, which was adjusted to pH 7.4 with NaOH) and incubated in secretion buffer containing ligands for 2 h for STC-1 cells and 1 h for colonic crypts at 37°C. Inhibitors were used as for signaling assays. Following incubation, cell supernatants were collected, and the cells were lysed with lysis buffer (0.25 g sodium deoxycholate monohydrate, 0.88g NaCl, 0.5mL Igepal, 80 mM Tris HCL, pH 8, 1 tablet of complete EDTA-free protease cocktail inhibitor (Roche)). Samples were analyzed for GLP-1 secretion via an established in-house radioimmunoassay

(Kreymann et al., 1987) using the NE1600γ scintillation counter (Thermo Electron Corporation). The GLP-1 antibody has 100% cross-reactivity with all amidated forms of GLP-1 but does not cross-react with glycine extended forms. The intra-assay coefficients of variation for GLP-1 were 5.6%. As a control of GLP-1 release in the presence of inhibitors, cells or colonic crypts in the absence of inhibitors that secreted GLP-1 equivalent or less than NaCl were excluded from further analysis. All experiments were conducted in duplicate for colonic crypts and triplicate for STC-1 cells and repeated at least 3 times.

Flow cytometry

Flow cytometry was used to quantitate internalization of FFA2 by measuring levels of receptor loss from the surface. Cells were fed live with M1 anti-FLAG antibody (20 min, 37°C) prior to treatment with ligands. Cells were then washed, lifted with PBS containing 2% FBS, centrifuged, and cell pellet washed with PBS and incubated with Alexa Fluor 488 secondary antibody (1h, 4°C). The fluorescence intensity of 10,000 cells were collected for each treatment and performed in triplicate using a FACS Calibur flow cytometer (BD Biosciences). Cells that were not exposed to any antibodies or secondary antibody alone were used for controls. All experiments were conducted at least three times.

Immunofluorescence and confocal imaging

Receptor imaging in live or fixed cells were conducted by incubating live cells with FLAG M1-antibody (F3040, Sigma, 1/500) (20 min, 37°C) and then with fluorescent secondary antibody (20 min, 37°C for live cell imaging) in phenol-red-free DMEM prior to agonist treatment. If inhibitors were used these were administered to the cells at appropriate time before ligand stimulation. To fix cells, cells were washed three times in PBS/0.04% EDTA to remove FLAG antibody bound to surface receptors prior to fixation with 4% paraformaldehyde in PBS (20 min), blocked with 2% FBS (1 h), permeabilized using 0.2% TritonX100, incubated with either anti-rabbit EEA1 (3288, Cell Signalling, 1/400) or APPL1 (3858, Cell Signalling, 1/400) for 1 h, washed and subsequently incubated with goat anti- mouse or rabbit Alexa Fluor secondary antibodies (Invitrogen) (1 h) at RT. Cells were washed again and mounted with Fluoromount-G (Thermo Fisher). Both live and fixed cells were visualized via a TCS-SP5 microscope (Leica) with a 63x oil-immersion objective and 1.4 numerical aperture (NA). Images were acquired using Leica LAS AF image acquisition software. Raw-image file were analyzed using ImageJ or LAS AF Lite (Leica) to measure endosomes diameter size or level of co-localization.

TIRFM

Cells were imaged using the Elyra PS.1 AxioObsever Z1 motorized inverted microscope with a sCMOS or EMCCD camera and an alpha Plan-Aprochromat 100x/1.46 Oil DIC M27 Elyra objective (Zeiss), with solid-state lasers of 488 nm, 561 nm and/642 nm as light sources. For live cell imaging, approximately 15 minutes prior to imaging, culture media was replaced with Opti-MEM reduced serum media supplemented with HEPES. Imaging was then carried out using a Zeiss Elyra PS.1 microscope controlled at 37°C with 5% CO_2. Time-lapse movies of whole cells were taken for 60 seconds, at 10 frames per second (fps) using Zen lite acquisition software. Fixed cells were prepared as for confocal imaging.

Statistical analysis

Data are represented as mean ± SEM. Mann-Whitney t-test, one-way ANOVA followed by Dunnett's post-test, or two-way ANOVA followed by Bonferroni post-test was used when comparing two groups, more than two groups or at least two groups under multiple conditions, respectively. Statistical significance was determined using GraphPad Prism. The number of samples (n) has been indicated for each figure panel. Differences were considered significant $p \leq 0.05$.

Supplemental References

Hoveyda, H. R., Fraser, G. L., Zoute, L., Dutheuil, G., Schils, D., Brantis, C., Lapin, A., Parcq, J., Guitard, S., Lenoir, F., et al. (2018). N-Thiazolylamide-based free fatty-acid 2 receptor agonists: Discovery, lead optimization and demonstration of off-target effect in a diabetes model. Bioorg Med Chem, 26, 5169-5180.

Kreymann, B., Williams, G., Ghatei, M. A. & Bloom, S. R. (1987). Glucagon-like peptide-1 7-36: a physiological incretin in man. Lancet, 2, 1300-4.

Maslowski, K. M., Vieira, A. T., Ng, A., Kranich, J., Sierro, F., Yu, D., Schilter, H. C., Rolph, M. S., Mackay, F., Artis, D., et al. (2009). Regulation of inflammatory responses by gut microbiota and chemoattractant receptor GPR43. Nature, 461, 1282-6.

Olken, N. M. & Marletta, M. A. (1992). NG-allyl- and NG-cyclopropyl-L-arginine: two novel inhibitors of macrophage nitric oxide synthase. J Med Chem, 35, 1137-44.

Roman, G., Vlahakis, J. Z., Vukomanovic, D., Nakatsu, K. & Szarek, W. A. (2010). Heme oxygenase inhibition by 1-aryl-2-(1h-imidazol-1-yl/1h-1,2,4-triazol-1-yl)ethanones and their derivatives. ChemMedChem, 5, 1541-55.
